# The eukaryome of African children is influenced by geographic location, gut biogeography, and nutritional status

**DOI:** 10.1093/femsml/uqad033

**Published:** 2023-07-20

**Authors:** Pascale Vonaesch, Vincent Billy, Allison E Mann, Evan Morien, Azimdine Habib, Jean-Marc Collard, Michel Dédé, Nathalie Kapel, Philippe J Sansonetti, Laura Wegener Parfrey, for the Afribiota Investigators, Laurence Barbot-Trystram, Robert Barouki, Alexandra Bastaraud, Jean-Marc Collard, Maria Doria, Darragh Duffy, B Brett Finlay, Serge Ghislain Djorie, Tamara Giles-Vernick, Milena Hasan, Bolmbaye Privat Godje, Jean-Chrysostome Gody, Francis Allen Hunald, Nathalie Kapel, Jean-Pierre Lombart, Alexandre Manirakiza, Synthia Nazita Nigatoloum, Laura Wegener Parfrey, Lisette Raharimalala, Maheninasy Rakotondrainipiana, Rindra Randremanana, Harifetra Mamy Richard Randriamizao, Frédérique Randrianirina, Annick Robinson, Pierre-Alain Rubbo, Philippe Sansonetti, Laura Schaeffer, Ionela Gouandjika-Vassilache, Pascale Vonaesch, Sonia Sandrine Vondo, Inès Vigan-Womas

**Affiliations:** Unité de Pathogénie Microbienne Moléculaire, Institut Pasteur, 25-28 Rue du Dr Roux, 75015 Paris, France; Departments of Botany and Zoology, and Biodiversity Research Centre, University of British Columbia, 3200-6270 University Boulevard, V6T1Z4 Vancouver, Canada; Departments of Botany and Zoology, and Biodiversity Research Centre, University of British Columbia, 3200-6270 University Boulevard, V6T1Z4 Vancouver, Canada; Departments of Botany and Zoology, and Biodiversity Research Centre, University of British Columbia, 3200-6270 University Boulevard, V6T1Z4 Vancouver, Canada; Unité de Bactériologie Expérimentale, Institut Pasteur de Madagascar, BP1274 Ambatofotsikely Avaradoha 101 Antananarivo, Madagascar; Unité de Bactériologie Expérimentale, Institut Pasteur de Madagascar, BP1274 Ambatofotsikely Avaradoha 101 Antananarivo, Madagascar; Laboratoire d’Analyse médicale, Institut Pasteur de Bangui, Avenue De Independence Bangui, 923 Central African Republic; Laboratoire de Coprologie Fonctionnelle, Assistance Publique- Hôpitaux de Paris, Hôpital Pitié-Salpêtrière, 47-83 Bd de l’Hôpital, 75013 Paris, France; Unité de Pathogénie Microbienne Moléculaire, Institut Pasteur, 25-28 Rue du Dr Roux, 75015 Paris, France; Departments of Botany and Zoology, and Biodiversity Research Centre, University of British Columbia, 3200-6270 University Boulevard, V6T1Z4 Vancouver, Canada

**Keywords:** human intestinal eukaryome, biogeography, fungi, protists, Sub-Saharan Africa, microbiota

## Abstract

Eukaryotes have historically been studied as parasites, but recent evidence suggests they may be indicators of a healthy gut ecosystem. Here, we describe the eukaryome along the gastrointestinal tract of children aged 2–5 years and test for associations with clinical factors such as anaemia, intestinal inflammation, chronic undernutrition, and age. Children were enrolled from December 2016 to May 2018 in Bangui, Central African Republic and Antananarivo, Madagascar. We analyzed a total of 1104 samples representing 212 gastric, 187 duodenal, and 705 fecal samples using a metabarcoding approach targeting the full ITS2 region for fungi, and the V4 hypervariable region of the 18S rRNA gene for the overall eukaryome. Roughly, half of all fecal samples showed microeukaryotic reads. We find high intersubject variability, only a handful of taxa that are likely residents of the gastrointestinal tract, and frequent co-occurrence of eukaryotes within an individual. We also find that the eukaryome differs between the stomach, duodenum, and feces and is strongly influenced by country of origin. Our data show trends towards higher levels of *Fusarium equiseti*, a mycotoxin producing fungus, and lower levels of the protist *Blastocystis* in stunted children compared to nonstunted controls. Overall, the eukaryome is poorly correlated with clinical variables. Our study is of one of the largest cohorts analyzing the human intestinal eukaryome to date and the first to compare the eukaryome across different compartments of the gastrointestinal tract. Our results highlight the importance of studying populations across the world to uncover common features of the eukaryome in health.

## Teaser

This study uses amplicon sequencing to unravel the intestinal eukaryome of almost 1000 African children and shows that it is influenced by geographic location, gut biogeography, and nutritional status.

## Introduction

The human gastrointestinal (GI) microbiome is a complex community comprised of bacteria, archaea, viruses, and eukaryotes (fungi, protists, and helminths). Many studies have mapped the bacterial microbiome across life stages, geographic locations, and health states and demonstrated its importance in health and disease (Yatsunenko et al. 2012, Luan et al. 2015, Castanys-Muñoz et al. 2016, Dominguez-Bello et al. 2019). Despite the clear contributions of some host-associated eukaryotes to human health, eukaryotes have been less often characterized than the bacterial members of the gut microbiome, particularly in large-scale studies (Parfrey et al. 2014, Stensvold and van der Giezen 2018, Mann et al. 2020). Hurdles include the lower biomass of eukaryotes in the gut (eukaryotes constitute < 0.1% of the total biomass of the microbiota; Qin et al. 2010), the smaller community studying eukaryotes, particularly outside of parasitology, technical difficulties arising from less well-curated databases (del Campo et al. 2020), and difficulty separating gut residents from eukaryotes introduced via diet (Suhr and Hallen-Adams 2015, Mann et al. 2020).

Studies using 18S rRNA targeted amplicon sequencing showed that protists and fungi, especially members of the *Saccharomycetales*, are the dominant eukaryotes in the human GI tract (Parfrey et al. 2014, Scanlan et al. 2018). Protists are a common part of the gut community (Stensvold et al. 2011a, b, 2020, Forsell et al. 2012, Roser et al. 2013, Parfrey et al. 2014, Krogsgaard et al. 2015, 2018, Jokelainen et al. 2017, Stensvold and van der Giezen 2018, Stensvold 2019, Mann et al. 2020) and are regularly detected in the feces of infants and toddlers (Jokelainen et al. 2017). In the past, studies describing the human eukaryome using specific Polymerase Chain Reaction (PCR) primers reported that the protist genera *Blastocystis* and *Dientamoeba* (Scanlan and Marchesi 2008, Velasco et al. 2011, Roser et al. 2013, El Safadi et al. 2014, Turkeltaub et al. 2015, Andersen and Stensvold 2016, Jokelainen et al. 2017) are widespread in healthy humans. Amoebae, especially *Entamoeba coli*, have been also described as commensal members of the human gut microbiome (ten Hove et al. 2007, Bruijnesteijn van Coppenraet et al. 2009, Stensvold et al. 2011b, Krogsgaard et al. 2018). Further, the presence of *Blastocystis* has been associated with sanitation levels, water source, and contact with animals or other infected humans (El Safadi et al. 2014, Beghini et al. 2017, Scanlan et al. 2018) and is generally reported at higher prevalence in low- and middle-income countries. Fungi are also commonly found in the intestinal microbiota of many different mammals, including humans (El Mouzan et al. 2017, Hallen-Adams and Suhr 2017, Nash et al. 2017, Auchtung et al. 2018, Lavrinienko et al. 2021, Boutin et al. 2021, Sun et al. 2021). In humans, fungi colonize the infant intestinal tract shortly after birth (Schei et al. 2017). Thus, there is consistent detection of eukaryotes in the intestinal microbiota of healthy subjects.

Recent cross-kingdom analyses show that fungi (Huseyin et al. 2017) and microeukaryotes (Stensvold 2019) are active participants in the GI ecosystem and influence health and disease through interactions with each other, other microbes, and the host. Microeukaryotes including *Blastocystis, Giardia, Entamoeba* (Verweij et al. 2004, ten Hove et al. 2007, Velasco et al. 2011), and a variety of fungi (Luan et al. 2015, Limon et al. 2019, Richard and Sokol 2019) have been linked to GI disease, antibiotic-associated diarrhea, and chemotherapy-induced enteric disorders (Stensvold and van der Giezen 2018), inflammatory bowel disease (IBD; El Mouzan et al. 2017, Sovran et al. 2018, Limon et al. 2019, Richard and Sokol 2019), and asthma (Arrieta et al. 2018, Goldman et al. 2018, Boutin et al. 2021) [reviewed in Huseyin et al. (2017)]. Further, the human mycobiome is changed in obesity (Mar Rodriguez et al. 2015) and fungal–bacterial interactions are perturbed in patients suffering of IBD (Sovran et al. 2018). Other microeukaryotes, such as *Giardia intestinalis* and *Entamoeba histolytica*, are pathogens that directly cause substantial morbidity and mortality (Roberts et al. 2013). Last, helminths, which are often considered pathogens, are well known to have an immunoregulatory function (Walsh et al. 2009, Broadhurst et al. 2012, Zaiss et al. 2015, Finlay et al. 2016, Gause and Maizels 2016, Giacomin et al. 2016, Ramanan et al. 2016) and the presence of several helminths has been associated with changes in the bacterial community (Walk et al. 2010, Cantacessi et al. 2014, McKenney et al. 2015, Zaiss et al. 2015, Giacomin et al. 2016). There is thus a clear link between alterations in the fungal and microeukaryotic community of the intestinal tract and disease.

In contrast to these observations, a growing number of studies show that protists such as *E. coli* and *Blastocystis* are common in healthy people (El Safadi et al. 2014, Krogsgaard et al. 2015, Andersen and Stensvold 2016, Beghini et al. 2017, Nieves-Ramirez et al. 2018), suggesting that they might also be important to maintain proper gut homeostasis (Audebert et al. 2016, Andersen and Stensvold 2016, Beghini et al. 2017) and are, thus indicators of a healthy gut ecosystem (Stensvold and van der Giezen 2018). Indeed, some of them, as an example *Blastocystis* (Krogsgaard et al. 2015) or *Dientamoeba* are even more common in healthy individuals than in comparison groups with immune mediated disease such as IBD (Andersen and Stensvold 2016, Beghini et al. 2017) and irritable bowel syndrome [reviewed in Stensvold and van der Giezen (2018)]. Further, colonization with helminths were virtually universal in human populations before the adoption of modern, highly sanitized lifestyles (Goncalves et al. 2003) and their drastic decrease in urban industrial populations has been discussed as a possible contributor to the concomitant rise of autoimmune disease (Rook 2012). Another recent observation is that coinfection with different eukaryotic pathogens can lead to reduced virulence in some conditions [reviewed in Venter et al. (2022)]. In this light, reduced diversity of helminths and other microeukaryotes (Parfrey et al. 2014) in industrialized countries may be altering the gut ecosystem directly through loss of species and indirectly by the associated loss of interactions.

Thus, the role of microeukaryotes in the intestinal tract seems to be complex and to depend on the particular GI ecosystem they inhabit.

There is clear evidence of geographic differences in the bacterial microbiome, mainly mediated through diet (De Filippo et al. 2010). Further, globalization and urbanization have been shown to be associated with a major loss in microbial diversity compared to a more traditional lifestyle (Clemente et al. 2015, Obregon-Tito et al. 2015, Smits et al. 2017, Jha et al. 2018, Pasolli et al. 2019). For the eukaryome, to date, most studies have been performed on a single geographic location. A small study in South Africa has shown that the mycobiome is affected by urbanization (Kabwe et al. 2020). A larger study in China, spanning several ethnicities and geographic regions showed very high variability across different geographic regions (Sun et al. 2021), mainly reflecting dietary habits and the urbanization gradient. For microeukaryotes, there are some geographic differences in the subtypes of *Blastocystis* (Alfellani et al. 2013). These studies clearly show the need to investigate the overall eukaryome make-up across different geographic locations.

So far, most studies assessing the microbiome focus on fecal samples as an overall read-out of the microbial intestinal community. There is, however, clear evidence for bacterial community changes along the GI tract (Vonaesch et al. 2018); there are likely similar changes in the eukaryotic community. The different segments of the GI tract have different roles and physiology, with nutrient absorption taking place mainly in the small intestine and fecal samples representing an overall read-out of the lower GI tract microbiota.

In this study, we aimed to characterize and compare the intestinal and fecal eukaryome in children aged 2–5 years living in two African countries: Madagascar and the Central African Republic (CAR). We then investigate the relationship between the eukaryome composition and clinical factors such as stunted growth, iron deficiency, and intestinal inflammation.

## Methods

### Study cohort, sample collection, metadata, and biobanking

This study was carried out in the context of the AFRIBIOTA project, a case-control study for stunting in children aged 2–5 years in two different study sites, Bangui, CAR and Antananarivo, Madagascar (Vonaesch et al. 2018). Recruitment took place between December 2016 and March 2018. In the context of this analysis, only children recruited in the community were included. Metadata including age, nutritional status, iron levels, hemoglobin, and socio-economic factors were collected using a standardized questionnaire. Complete blood count, C-reactive protein (CRP), and ferritin levels were measured at the Clinical Biology Center (CBC) of the Institut Pasteur de Madagascar and the Laboratoire d'Analyse Médicale at the Institut Pasteur de Bangui within 4 h after blood collection. Ferritin levels were corrected for systemic inflammation as described in (Thurnham et al. 2010). Hemoglobin values were adjusted for altitude as described in Centers for Disease Control (CDC; 1989) and Sullivan et al. (2008), and anemia was defined as less than 110 g/l according to WHO criteria (Onis 2006, OMS 2011). All participants received oral and written information about the study and legal representatives of the children provided written consent to participate in the study. The study protocol for AFRIBIOTA has been approved by the Institutional Review Board of the Institut Pasteur (2016–06/IRB) and the National Ethical Review Boards of Madagascar (55/MSANP/CE, 19 May 2015) and CAR (173/UB/FACSS/CSCVPER/16). Detailed inclusion and exclusion criteria and recruitment procedures are described elsewhere (Vonaesch et al. 2018); importantly, children with severe acute disease were excluded, as were children that had recently taken antibiotics (Vonaesch et al. 2018). Based on median height of the WHO reference population (Onis 2006, World Health Organization 2007), the children were classified in three groups: severe stunting (height-for-age z-score ≤ −3SD), moderate stunting (height-for-age z-score between −3SD and −2SD), and not stunted (height-for-age z-score ≥ −2SD). Caregivers were instructed to collect feces in the morning before coming to the hospital. Gastric and duodenal samples were collected using a pediatric nasogastric tube (Vygon, France), and were only collected for stunted children (ethical constraints). The procedure was put in place for studying the bacterial community of the small intestine, which was suspected, and later shown, to be implicated in the pathophysiology of stunting (Vonaesch et al. 2018, 2022). We, thus had the unique opportunity to analyze the intestinal eukaryome from these samples. Once the gastric, duodenal, or fecal samples were collected, they were aliquoted, frozen at −20°C and transferred the same day to a −80°C freezer (Bangui), or directly snap-frozen in liquid nitrogen and then transferred to a −80°C freezer (Antananarivo). DNA extraction was performed on site in Antananarivo and Bangui and extracted DNA was shipped on dry ice. Biobanking and sample distribution was performed by the Unité de Bactériologie Expérimentale, Institut Pasteur de Madagascar, the Laboratoire d'Analyse Médicale, Institut Pasteur de Bangui, and the Clinical Investigation and Access to BioResources Platform (ICAReB) at the Institut Pasteur, Paris. The STORMS checklist for this study is included as File S1 (Supporting Information).

### DNA extraction and sequencing

Gastric, duodenal, and fecal samples were extracted by commercial kits (QiaAmp cador® Pathogen Mini or cador® Pathogen 96 QIAcube® HT Kit, Qiagen, which are kits using the same chemistry for manual or automatic extraction) following the manufacturer’s recommendations with an additional bead-beating step to increase mechanical disruption as described in Vonaesch et al. (2018). DNA extraction was compared between the two sites using bacterial ZymoBiomics community standards (Zymobiomics, D6300) and DNA contamination was assessed using parallel processed negative controls (molecular grade water). Samples were stored at −80°C until sequencing. Extracted DNA samples were shipped on dry ice to a commercial provider where library generation and sequencing was performed (Microbiome Insights, Canada).

For ITS2 sequencing, the primers ITS4 (5′-CCTCCGCTTATTGATATGC-3′) and fITS7 (5′-CCGTGARTCATCGAATCTTTG-3′) were used (Ihrmark et al. 2012). Primer choice was based on Nash et al. (2017). PCR conditions were identical to the ones described in Gweon et al. (2015). 18S primers TAReuk454FWD1 (5′-CCAGCASCYGCGGTAATTCC-3′) and TAReukREV3 (5′-ACTTTCGTTCTTGATYRA-3′) were chosen to preferentially amplify protists (Stoeck et al. 2010, Maritz et al. 2017). In brief, library synthesis and amplification were performed using Phusion High-Fidelity PCR Master Mix (Thermo Scientific, catalogue #F-531S) in a 20 μl reaction volume, and a two-step PCR amplification strategy according to the following protocol: 98°C for 30 s, 10 cycles of 98°C for 10 s, 53°C for 30 s, 72°C for 30 s; and then 25 cycles of 98°C for 10 s, 48°C for 30 s, 72°C for 30 s, and ending at 72°C for 10 min. Sequencing was performed on an Illumina MiSeq using the 300-bp paired-end kit (v.3). Only high quality forward reads (R1) were used for the analysis.

For a subset of the data, a second library using the same 18S rRNA V4 amplicon primers (TAReuk454FWD1 and TAReukREV3) and a peptide nucleic acid primer to block amplification of mammalian sequences (5′-TCTTAATCATGGCCTCAGTT-3′) (Mann et al. 2020) was performed at the University of British Columbia, Vancouver, Canada using identical PCR conditions. The mammalian blocking primer was designed to reduce the amplification of human reads and thus increase sequencing depth for nonhuman eukaryotic reads (Mann et al. 2020). Sequencing of this second data set was performed at Dalhousie University on a MiSeq Illumina sequencer using 300+300 bp paired-end V3 chemistry as described in Kozich et al. (2013).

### Bioinformatic analysis

#### 18S rRNA V4 datasets

An average of 48 893 (minimum: 1; maximum: 369 607; total: 42 879 181) reads were generated for the 18S dataset.

Demultiplexed reads were obtained from the two sequencing facilities and were processed into amplicon sequence variants (ASVs) using the DADA2 pipeline (Callahan et al. 2016) with a minimum sequence length of 150 and maximum expected error of 8. Overall, for the 18S rRNA dataset using no blocking primer, we obtained 694 978 clean sequences after running the Dada2 pipeline and filtering out any nonmicroeukaryotic reads such as vertebrates or plants. On average, we had 2632 sequences per sequencing-positive sample (i.e. with any sequences amplified; median: 1638; minimum: 0; maximum: 21 327). Taxonomy for the 18S dataset was assigned in a multistep process. First, taxonomy was assigned using DADA2 and with the integrated tool SINA (Pruesse et al. 2012) and against the SILVA database (version 128) after which any ASV that could not be assigned a taxonomy was compared to the PR2 database (version 4.11.1) (Guillou et al. 2013). ASVs present in only one sample and at a relative abundance of less than 0.1% of the total dataset were removed. Samples with fewer than 5000 reads were removed from downstream analyses.

An average of 30 203 (minimum: 289; maximum: 267 972; total: 9 393 270) reads were generated for the 18S dataset using a blocking primer. Overall, for the 18S rRNA dataset using the blocking primer, we obtained 3 415 973 clean sequences after running the Dada2 pipeline and filtering out any nonmicroeukaryotic reads such as vertebrates or plants. On average, we had 6222 sequences per sequencing-positive sample (i.e. with any sequences amplified; median: 983; minimum: 2; maximum: 84 623). ASVs for this dataset were generated in an identical manner to the full 18S dataset and samples with fewer reads than 500 were removed from downstream analyses. 18S sample processing files can be found at https://github.com/Parfreylab/afribiota.

Taxonomy was refined by cross-referencing with the other databases and by placement in phylogenetic trees for key taxa as described below. Suspected taxonomic misannotations were checked using BLASTn and the NCBI NT database (McGinnis and Madden 2004, Ye et al. 2006, Johnson et al. 2008) and the taxonomic string assignment for *Entamoeba* and *Saccharomycetales*, which are erroneous in the Silva database, were corrected manually. All corrections to the original taxonomy file are listed in Table S1 (Supporting Information). Code for the bioinformatic analysis is available at https://github.com/Parfreylab/afribiota.

#### ITS2 dataset

An average of 57 170 (minimum: 24, maximum: 339 136) sequences per sample were generated for the ITS2 dataset. ASVs were generated using the DADA2 pipeline with a minimum sequence length of 50 and maximum expected error set to 6 and 8 for the forward and reverse reads, respectively. After dereplication, ASVs comprised of fewer than 50 reads or those with less than 0.1% overall relative abundance in the full dataset were removed. On average, we obtained 40 576 clean sequences per sample for the ITS2 dataset after running the Dada2 pipeline (minimum: 0; maximum: 217 506). Samples with fewer than 5000 reads were removed from downstream analyses. Taxonomy was assigned using the UNITE database (version 8.0) (Koljalg et al. 2005). Fungal ASVs that could not be assigned beyond the kingdom level using the UNITE database (36.7% of the total dataset) were run through BLAST against the NCBI NT database but no further taxonomic information could be resolved and the sequences were thus just assigned at Kingdom level. All code for sample processing is available at https://github.com/parfreylab/afribiota. Rarefaction curves for all datasets are given in Figure S1 (Supporting Information). All raw sequencing data is deposited in ENA, accession number PRJEB57073.

### Annotation of taxa as environmental, gut-associated, or potentially gut-associated taxa

Taxa were individually assessed and screened for human-association using expert knowledge and Google search. Taxa were classified as gut-associated if the taxon has been associated with human infection or repeatedly described to be part of the eukaryotic microbiome in peer-reviewed scientific journals. Taxa were classified as possibly gut-associated if the taxon has been associated with human infection, but in very few reports or cases. Taxa were classified as environmental if they are known plant parasites, are known to inhabit aquatic environments or soil, or are known to be associated with nonhuman eukaryotes such as insects. If there was no hit for the taxon in the Google search or if the taxonomic level did not allow distinguishing the taxon enough to make a claim about the most likely origin or association, taxa were classified as from unknown origin.

### Construction of phylogenetic trees

Taxonomy was refined by phylogenetic analysis for key eukaryotes by constructing backbone trees and then placing ASVs within these trees. A total of five backbone trees were generated, one for *Blastocystis, Entamoeba, Trichostomatia, Diplomonada*, and *Trichomonada*. For each backbone tree, we retrieved available sequences from the SILVA (v.132), PR2 (v.4.12.0) and NCBI databases. Sequence accession numbers used for the backbone trees are presented in Table S2 (Supporting Information). We also used out-group taxa selected based on the literature to root the trees. Sequences were first sorted and clustered at 99% similarity using usearch (v.8.1.1831_i86linux32) or vsearch (v.2.15.1) (Rognes et al. 2016). Sequences were then aligned using mafft (v.6.814b or v.7.475) (Katoh et al. 2009) and trimmed using trimAl (v.1.2rev59 or v.1.4.1) (Capella-Gutierrez et al. 2009), and the alignment inspected using aliview (v.1.26) (Larsson 2014). A maximum-likelihood phylogenetic tree was then constructed using a GTR model and 100 bootstrap replicates with RAxML (v.8) 95 (Stamatakis 2015, Kozlov et al. 2019). ASVs retrieved from the Afribiota dataset were then placed into the resulting backbone tree using SINA (v.1.7.1) (Pruesse et al. 2012). The annotation of 102 ASVs was then updated based on the backbone trees (Table S1, Supporting Information). *Blastocystis* ASVs were further curated based on the alignment and tree. Two ASVs fall outside of known subtypes (DALASV146 and DALASV723); DALASV146 matches at 100% similarity with accession OM057456.1, which is sampled from Hoolock Gibbon. Subtypes ST1, ST2, and ST3 were represented by many ASVs and were further collapsed into subclusters if sequences were >97% similar and formed a clade in the phylogenetic tree (Table S1 and Figure S4, Supporting Information). Subclusters did not show one to one correspondence with *Blastocystis* allele designations because the sequence fragment here is shorter than the fragment used for allele designation and missing informative sites; for this reason we refer to subclusters by the ASV# of the ASV with highest relative abundance rather than allele. The most similar allele for each ASV within a subcluster was determined using the *Blastocystis* Public databases for molecular typing and microbial genome diversity (Stensvold et al. 2012) and reported along with ASV in Table S1 (Supporting Information). Conda environment and scripts used to generate the *Entamoeba* tree are available at https://github.com/aemann01/afribiota and the ones to generate the *Blastocystis, Trichostomatia, Diplomonada*, and *Trichomonada* trees at https://github.com/parfreylab/afribiota.

### Measurement of fecal markers of inflammation

Fecal calprotectin concentration was assayed in duplicate by a ‘sandwich’ type enzyme-linked immunosorbent assay, which uses a polyclonal antibody system (Calprest; Eurospital, Italy). The assay was performed according to the manufacturer’s instructions yielding a measurement range of 15–5000 µg/g. Fecal α1-antitrypsin was measured using an immune-nephelemetric method adapted on the BN ProSpec system (Siemens, Germany). Briefly, stool samples were diluted 1:5 in 0.15 M NaCl then shaken vigorously by the mean of a vortex until complete homogenization. The homogenate was centrifuged at 10 000 *g* for 15 min at 4°C and the supernatant was used for analysis, which was performed at two different dilutions (1:5 and 1:500 final dilutions) to avoid any prozone phenomena. Using this method, the range of measurement was 0.01–20 mg/g (Rodriguez-Otero et al. 2012).

### Identification of microeukaryotes by microscopy

The identification of parasites was performed on a subset of subjects, which provided enough stool samples according to methods reported previously (Habib et al. 2021). In short, fecal samples were examined microscopically using the Merthiolate–Iodine–Formaldehyde (MIF) and Kato-Katz (KK) techniques for helminths and the direct smear method (mounting without colouring) for protozoans according to standard techniques. The analyses were performed at the Medical Center and the Experimental Bacteriology Unit of the Institut Pasteur de Madagascar and the Institut Pasteur de Bangui and validated by a medical doctor for diagnostic purposes.

### Biostatistical analysis

The final, filtered dataset with >5000 sequences per sample comprised the following: for the 18S rRNA dataset without human blocking primers 33 gastric samples, 53 duodenal samples, and 464 fecal samples, for the 18S rRNA with human blocking primer 23 duodenal samples, 241 fecal samples and for the ITS2 dataset 158 gastric samples, 145 duodenal samples, and 315 fecal samples. Biostatistical analyses were performed using R, version 3.4.1 and the R packages Phyloseq (version 1.22.3) (McMurdie and Holmes 2013), microbiome (version 1.0.2) (Callahan et al. 2016), vegan (version 2.4–6) (Dixon 2009), DESeq2 (version 1.18.1) 103 (Anders and Huber 2010, Anders et al. 2013), and ggplot2 (version 3.3.0) (Journal of Statistical Software, 2010). For the 18S dataset, taxa without annotation beyond the kingdom level were excluded from the final analysis because many were misannotated bacterial sequences. Alpha diversity was quantified using the combined Shannon index using rarefied data including singletons. All other analyses were performed on datasets with singletons filtered out. Beta diversity was quantified using the Bray–Curtis dissimilarity index for data of relative abundance and the Jaccard index for presence–absence datasets (Bray and Curtis 1957). The Jaccard index was preferred over the Sorensen index to have equal weight for all taxa regardless of their prevalence across the dataset. Differences of diversity tests between samples were performed using nonparametric multivariate analysis of variance (PERMANOVA) with the function ‘adonis’ in the R package vegan (Dixon 2009) or using an iterative, logistic regression. *P*-values were corrected for multiple comparisons using the Benjamini–Hochberg procedure. Multivariate analyses of differentially abundant taxa as well as the presence of given taxa were performed on combined samples from both countries as well as on data from each country independently. Multivariate models were corrected for gender, age (in months), and country of origin and stratified on sample type, then on country of origin. For fecal samples, the multivariate models were also corrected for calprotectin levels as a measure of intestinal inflammation. Metadata, ASV tables and taxonomy tables can be found in the Appendix (Supporting Information) and the code is available at https://github.com/parfreylab/afribiota.

## Results

### Description of study population and the presence of eukaryotic reads

We analyzed a total of 1104 biological samples from a cohort of children from Madagascar and CAR with two primer sets to investigate the fungal (ITS2 primers) and microeukaryotic (18S rRNA gene primers) components of the gut microbiome. The sample filtering workflow is summarized in Figure S3 (Supporting Information). The overall characteristics of the children included in the study are given in Table S3 (Supporting Information) and the distribution of the main clinical variables in Figure S2 (Supporting Information).

Host, dietary, and environmental taxa were frequently amplified with 18S primers, as is common for studies of the mammalian gut (Mann et al. 2020). Removing sequences corresponding to plants or vertebrates from the 18S dataset left 464 fecal samples and 53 duodenal samples that had microeukaryotic reads (66% and 28%, respectively) (Figure S3A, Supporting Information). We also amplified a subset of 312 samples with 18S primers plus a mammalian blocking primer; 241 fecal samples (92%), and 23 duodenal samples (72%) were above the threshold of 500 sequences set by the background (negative control; Figure S3B, Supporting Information). After filtering out plant and vertebrate reads, the microeukaryotic reads were very low in the duodenal and gastric samples. They were a median of 99 reads in the duodenal samples and nine reads in the gastric samples using no blocking primer compared to a median read count of 988 for fecal samples and 49 reads in the duodenal samples and 2886 reads in fecal samples in the dataset using a blocking primer. Thus, for the two 18S datasets, the statistical analyses were therefore only performed for the fecal samples. Amplification of ITS and 18S was uneven across samples, so we asked whether samples with reads above threshold levels were correlated with country of origin or clinical variables. There were significantly more microeukaryotic positive samples in Madagascar compared to Bangui (18S dataset with a blocking primer: *P* < .001 and without a blocking primer: *P* = .014). Further, there was a weak trend to more microeukaryotic positive samples in older children compared to younger children (18S dataset with a blocking primer *P* = .38, 18S dataset without a blocking primer: *P* = .026). Anemia in the fecal 18S dataset using a human blocking primer was the only clinical variable significantly associated with the presence of microeukaryotic reads. The dataset generated with a blocking primer yielded higher diversity and more eukaryotes likely to be gut residents (Figure S5, Supporting Information). We, thus focus in our analysis on this dataset and present results without the blocking primer in the supplement.

In the fungal ITS2 dataset 95% of the gastric samples sequenced (158 samples), 77% of the duodenal samples sequenced (145 samples), and 45% of the fecal samples sequenced (315 samples) had ITS2 (fungal) reads above the threshold of 5000 reads set by the negative control (Figure S3C, Supporting Information). Gastric and duodenal samples had a higher prevalence of fungi-positive samples compared to feces (*P* < .0001). The prevalence of samples with fungal reads in Madagascar (43%) was significantly higher compared to Bangui (33%; *P* = .03) and there were also more fungal reads per sample in Madagascar compared to Bangui (*P* = .03). The presence of fungal reads was not significantly associated with any of the clinical parameters measured in the duodenum or feces.

Thus, our data shows that most of the human samples contain eukaryotic reads and that the presence of an intestinal eukaryome is not strongly influenced by clinical parameters but is influenced by the children's country of residence and age.

### The fecal eukaryome of African children is dominated by *Blastocystis* and fungi

**Figure 1.**
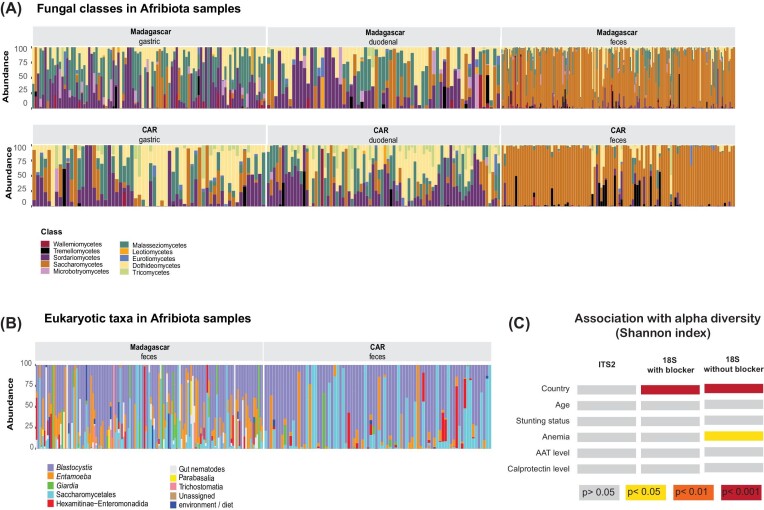
Composition of the Afribiota samples as revealed by ITS2 sequencing targeting fungi **(A)**, the broader 18S primers using a mammalian blocker **(B)** as well as association of the overall diversity with different clinical outcomes **(C)**. Significant association with the Shannon index as a measure of alpha diversity were assessed using a Wilcoxon rank-sum test. The colour code illustrating the degree of significance of the association is given on the bottom of the figure.

Characterizing the eukaryome reveals a handful of fungi and microeukaryotes that are prevalent across populations, while the overall eukaryome is low diversity and the distribution of most taxa patchy across individuals and populations (Figure 1A and B; Figure S8A, Supporting Information). After filtering for low abundance and low-prevalence taxa we recovered 219 ASVs in the ITS dataset of which 99 (45%) are unassigned at species level and 48 (22%) at phylum level.

After filtering for low-prevalence taxa (present in less than 50 sequences and less than 0.01% of the samples) and removing plants and vertebrates (18S rRNA dataset), we recovered 57 ASVs in the 18S rRNA dataset without using a mammalian blocking primer, and 127 ASVs in the reduced 18S rRNA dataset using a mammalian blocking primer.

As expected, for the ITS2 dataset, most of the taxa belong to the phylum Ascomycota or Basidiomycota. Overall, we identified two different fungal classes, 23 different orders, 41 different families, 60 different genera, and 78 different species (Tables S5–S10, Supporting Information). We identified a suite of eukaryotes spanning a broad taxonomic range that include many protists and helminths that are familiar from parasitology textbooks (Roberts et al. 2013), such as *Entamoeba, Giardia, Trichuris*, and *Ascaris* (Figure 3). The list of taxa detected is very likely an incomplete catalog of the microeukaryotic diversity in this cohort due to rather low sequencing depth and because many microeukaryotes are found at low frequency. We assessed whether using a mammalian blocking primer consistently increased the recovered diversity across microeukaryotic taxa by analyzing a subset of 100 fecal samples that were amplified with and without the mammalian blocking primer and compared taxon prevalence (Figure S5, Supporting Information). The mammalian blocking primer yielded higher sensitivity and diversity for protist genera, but depressed detection and prevalence of fungal and helminth genera (Figure S5, Supporting Information). We detected higher diversity of ASVs overall using the mammalian blocking primer (Shannon Index, *P* < .001; Figure S5, Supporting Information). This was mediated by both higher richness (Chao1, *P* < .001) and higher evenness of the community structure (Inverse Simpson, *P* < .001). Thus, the rest of the analysis is focused on the dataset using mammalian blocking primer as it is most representative of the diversity in the gut eukaryome and particularly for protists. Results on the slightly larger dataset not using mammalian blocking primers are presented in the supplementary data (Figures S6, S8–S10, S13, Tables S8, S9, and S19, Supporting Information).

We found two genera to be present in at least 50% of samples in the 18S dataset: *Blastocystis* and *Entamoeba*. Using mammalian blocking primer allowing for preferential detection of protists, we detected *Blastocystis* to be present in 75% of all fecal samples analyzed. Stratified by country, 50% of all samples from Madagascar amplified *Blastocystis* and *Entamoeba* and 50% of all samples from CAR *Blastocystis*.

In the dataset using a blocking primer, there were no consistent trends to co-occurrence or coexclusion of any of the eukaryotes with each other at lower taxonomic level (Figures S6A–C, Supporting Information). However, there were clear negative correlations between fungi and protozoa/helminths at higher taxonomic level (Figures S6D–G, Supporting Information). The same trends were observed in the dataset not using a mammalian blocker (data not shown). Several co-occurrences and coexclusions were observed in the fungal ITS2 dataset, yet often with weak associations (Figure S7, Supporting Information). There was also extensive coexistence of different *Blastocystis* subtypes within a single individual (Table S4, Supporting Information).

In summary, our data shows that the eukaryome of African children shows a high intersubject variability and is dominated by the protists *Blastocystis* and *Entamoeba* and different fungi.

### The mycobiome of African children is varied and dominated by members of *Saccharomyces*

**Figure 2.**
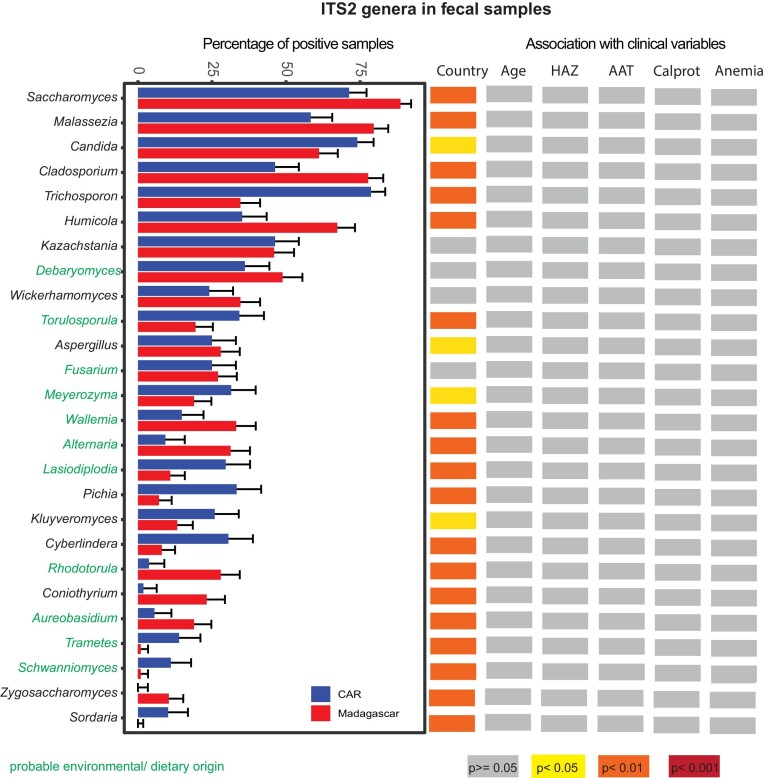
Differences in the fecal mycobiome in relation to geographic location and different clinical outcomes. Samples were considered to be positive for a given genus if they had at least a single reads relating to this genus. Genera indicated in green are of probable environmental origin. Groups were compared using the Pearson chi-squared test and Benjamini–Hochberg correction for multiple testing. The colour code illustrating the degree of significance of the association is given on the bottom of the figure.

The fecal mycobiome was largely dominated by Ascomycota (Figure 2) and more precisely by members of the group *Saccharomycetales* (average rel. abundance 74%; Tables S5 and S6, Supporting Information). There were very few fungi conserved on the lower taxonomic level. We detected five fungal genera to be present in at least 50% of all fecal samples and with a relative abundance of at least 0.001%: *Humicola, Malassezia, Cladosporium, Candida*, and *Saccharomyces* and two fungal species: *Humicola grisea* and *Malassezia restricta*.

Thus, our data highlights a dominance of Ascomycota in the GI tract of African children with members of the *Saccharomycetales* as the main constituents of the fecal mycobiome.

### The fecal eukaryome of children is strongly influenced by the country of residence

**Figure 3.**
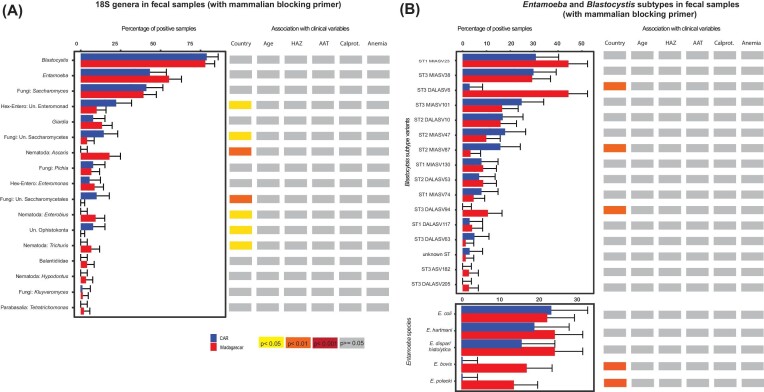
Differences in the fecal microeukaryome in relation to geographic location and clinical variables. **(A)** All genera as detected by 18S sequencing using a mammalian blocking primer and **(B)** split in different *Blastocystis* clusters or *Entamoeba* species based on phylogenetic trees. Samples were considered to be positive for a given genus if they had at least a single sequence relating to this genus. Groups were compared using the Pearson chi-squared test and Benjamini–Hochberg correction for multiple testing. The colour code illustrating the degree of significance of the association is given on the bottom of the figure.

**Figure 4.**
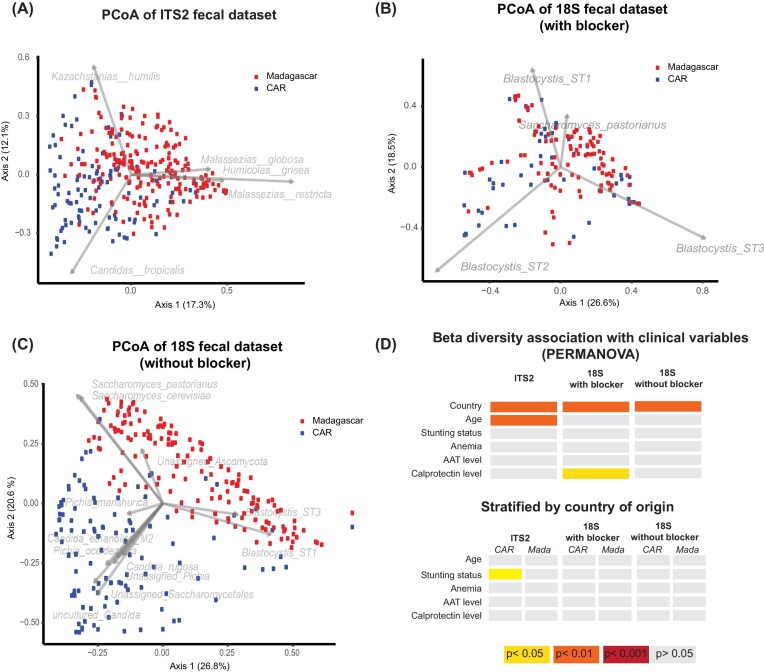
Differences in the microeukaryome (18S dataset) based on country of origin. PCoA plot based on the normalized Bray–Curtis dissimilarity index (log10) of the fecal dataset iteratively rarefied to 1000 microeukaryotic reads **(A)** with the ITS2 primers targeting fungi (CAR: *n* = 100, Madagascar: *n* = 202), **(B)** with a mammalian blocker using 18S primers (CAR: *n* = 54, Madagascar: *n* = 99), **(C)** without a mammalian blocker using 18S primers (CAR: *n* = 110, Madagascar: *n* = 154). Samples from CAR are coloured in blue, samples from Madagascar in red. **(D)** Association of different clinical factors with Beta Diversity using a Permanova analysis on dispersion. The colour code is given in the figure.

Overall, our data reveals that the fecal eukaryome of African children is influenced by country of residence (Figure 1C–4; Figures S8B, S9B–D, S10, Supporting Information). For all amplicon datasets, country of origin significantly contributes to the overall beta diversity when assessing both relative abundance (Bray–Curtis dissimilarity index; Figure 4D) and presence–absence (Jaccard index; Figures S9B–D, Supporting Information). Further, the prevalence of several taxa was significantly different between the two countries in a bivariate analysis (Figure 2 and 3; Figure S7B, Supporting Information). The relative abundance of several of these taxa remained significantly associated with the country of origin in a multivariate model correcting for sequencing run, total fungal reads and intestinal inflammation (Figure S10, Supporting Information).

In the 18S dataset without a mammalian blocker, prevalence of *Ascaris, Trichuris, Entamoeba*, and *Blastocystis* was slightly higher in Madagascar while *Candida, Pichia*, two different groups of unassigned *Saccharomycetales*, and one unknown enteromonad were more prevalent in CAR (Figure S8B, Supporting Information). When correcting for sequencing run, total microeukaryotic reads, and intestinal inflammation (alpha-antitrypsin levels and calprotectine levels), the two genera of unassigned *Saccharomycetales, Pichia, Entamoeba* and *Candida* remained significantly associated with the country of origin of the full dataset of fecal samples. In the reduced dataset using a mammalian blocking primer, we further found *Entamoeba polecki* to be significantly more prevalent in Madagascar compared to CAR (FDR = 0.007) in a multivariate model correcting for anemia status, intestinal inflammation, age and sequencing depth. Differences in alpha diversity according to the country of origin were visible in the 18S dataset but not in the ITS2 dataset (Figure 1C). The differences in microeukaryotes across taxa was further confirmed on a subset of samples and microeukaryotes using microscopy-based approaches (Table S19, Supporting Information).

Thus, our data show a clear influence of the country of origin on the eukaryotic community of fecal samples.

### High genetic diversity of *Blastocystis* and frequent mixed colonization

In the dataset sequenced using a mammalian blocking primer, 78% of all fecal samples had at least one subtype of *Blastocystis* detected in their feces (189/241). The most prevalent subtype detected was ST3 (53% prevalence; 128/241), closely followed by ST1 (46%; 111/241) and ST2 (33%; 79/241; Tables S6–S9, Supporting Information). Of the *Blastocystis* positive samples, 49% had only one *Blastocystis* subtype detected by 18S amplicon sequencing and 51% displayed mixed colonization (Table S4, Supporting Information). Overall, there was a slightly higher prevalence of *Blastocystis* ST3 in Madagascar compared to CAR (FDR = 0.05). *Blastocystis* subtypes were further divided into subclusters using phylogenetic analysis (Figure 3B; Table S1, Supporting Information). Several of the subclusters showed a different distribution according to the country of origin of the children: There was a higher prevalence of subclusters ST3-DALASV6 and ST3-DALASV94 in feces from Malagasy children compared to CAR children. We observed further a lower prevalence of subcluster ST2-MIASV87 in Madagascar compared to CAR (Figure 3B).

Together, these results indicate that children frequently have mixed colonization of distinct *Blastocystis* subtypes and genetic variability is high within *Blastocystis* subtypes. Some genetic variants (subclusters) show country-specific differences in prevalence.

### Different species of *Entamoeba* are part of the intestinal eukaryome

All sequences from *Entamoeba* were placed in a phylogenetic tree (Figure S4, Supporting Information) to refine the classification at species level. We detected five *Entamoeba* species: *E. coli, E. dispar/histolytica* (the ASVs detected there are all more similar to the nonpathogenic *E. dispar*, though it is not possible to confidently distinguish these species by amplicon sequencing), *E. polecki, E. bovis*, and *E. hartmanni. Entamoeba polecki*, previously known as *Entamoeba chattoni*, was detected in 8% of samples (20/242). We detected *E. dispar/histolytica* in 21% of all samples (50/242), *E. coli* in 22% (54/242), *E. hartmanni* in 22% (53/242), *E. polecki* in 8% (20/242), and *E. bovis* in 10% (25/242). Roughly, one-fourth of the *Entamoeba* positive samples had more than one *Entamoeba* species present. Further, for both, *Blastocystis* and *Entamoeba*, we saw a negative correlation with the relative abundance of fungi and a positive correlation in between *Blastocystis* and *Entamoeba* (Figure S6, Supporting Information).

In conclusion, our data shows the presence of different species of *Entamoeba* in fecal samples with pronounced country-specific differences in the prevalence of specific subtaxa. Most samples show only a single *Entamoeba* species at the time.

### Stunting is associated with altered abundance of certain members of the eukaryome

We next assessed if different clinical factors, including anemia, stunting status, and environmental enteric disease (measured through the inflammatory markers fecal calprotectin and alpha-1-antitrypsin) are associated with significant changes in the eukaryome composition in the GI tract (Figure 2 and 3; Figure S7B, Supporting Information). Overall, clinical factors contributed only marginally to the beta diversity (Figire 4D). Further, there was little influence of these clinical parameters on alpha diversity (Figure 1C), except for anemia and calprotectin levels, which contributed marginally to the alpha diversity of the 18S dataset using mammalian blockers. In a bivariate model, prevalence of *Ascaris, Trichuris*, and *Saccharomycetales* was associated with anemia (Figure S8, Supporting Information). However, no specific taxa were consistently associated with anemia, alpha-1-antitrypsin, or calprotectin levels in a multivariate analysis assessing for relative abundance of the reads in the 18S nor the ITS2 datasets (data not shown).

We then tested for associations between taxa prevalence and relative abundance and stunted growth in the ITS and 18S datasets (Figure S13, Supporting Information). In the ITS dataset the relative abundance of one taxon— *Fusarium equiseti*—was associated with stunted growth in CAR in a bivariate analysis assessing stunted growth as a categorical variable (corrected *P* = .03) and in a multivariate analysis (DeSeq2) that corrected for age, sequencing run and total fungal reads (*P* = .001). *Fusarium equiseti* was also found with higher prevalence in stunted children compared to nonstunted controls in a multivariate logistic regression model (*P* = .03) correcting for country of origin, calprotectin levels, sequencing run, total fungal reads, and gender. Together, these data suggest that there might be more *F. equiseti* in stunted children compared to nonstunted controls.

In the 18S dataset using the mammalian blocking primers, there was a trend of reduced *Blastocystis* relative abundance in stunted children compared to nonstunted controls. This was observed in the overall dataset (including both study sites) performing a bivariate correlation analysis (FDR = 0.07). Stratifying the analysis by country, this trend was observed in bivariate analysis using stunting either as a categorical variable in Antananarivo (FDR = 0.08), as a continuous variable (FDR = 0.016), but not in CAR. The association with *Blastocystis* was not significant in a multivariate model correcting for intestinal inflammation, anemia, age, and gender, which are known modulators of the microbiota. The same trends were observed in the 18S dataset not using a mammalian blocking primer. Further, in both 18S datasets, several members of the *Saccharomycetales* were consistently associated with stunting in Bangui, CAR.

Thus, our data indicates that there are consistent trends for lower *Blastocystis* and *Saccharomycetales* levels and higher *F. equiseti* levels in stunted children in both study sites. The associations are independent of intestinal inflammation and/or anemia.

### The human eukaryome differs along the GI tract

We examined the mycobiome and eukaryome along the GI tract. Data for the eukaryome of the upper GI tract are sparse; most of the microeukaryotic reads in the gastric and duodenal samples belong to host, dietary, and environmental sources, while gut residents, such as *Entamoeba* and *Blastocystis*, were sporadically observed. After filtering out vertebrate, arthropod, and plant reads (which are likely of dietary origin) there were only few gastric and duodenal samples with microeukaryotic reads with and without the mammalian blocking primer (Figure S3A and B, Supporting Information).

**Figure 5.**
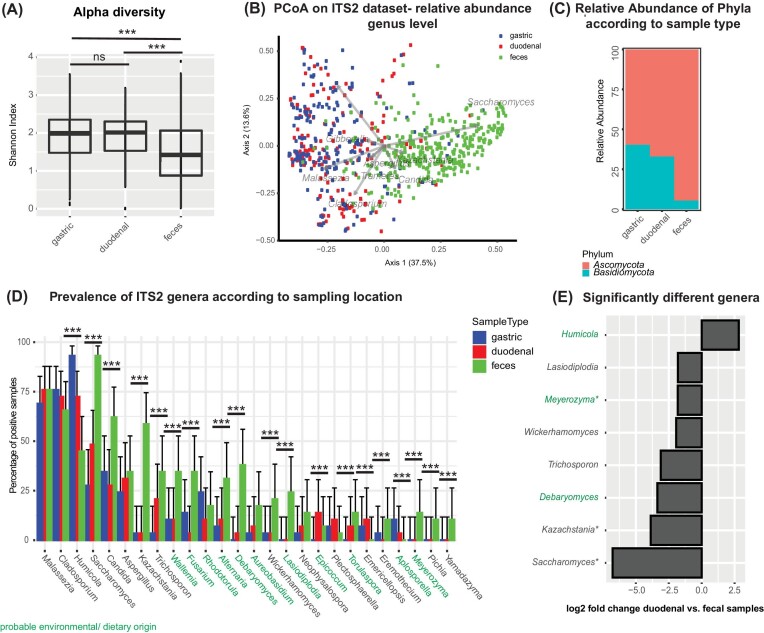
Differences in the mycobiome along the GI tract. **(A)** Alpha diversity in the different compartments as measured by the Shannon index. **(B)** PCoA plot based on the normalized Bray–Curtis dissimilarity index (log10) of the dataset iteratively rarefied to 5000 fungal sequences (gastric: *N* = 148, duodenal: *N* = 132, and feces: *N*=299). **(C)** Relative abundance of the different phyla according to sample type. **(D)** Differences in the mycobiome in relation to sampling location along the GI tract. Samples were considered to be positive for a given genus if they had at least a single sequence relating to this genus. Groups were compared using the Pearson chi-squared test and Benjamini–Hochberg correction for multiple testing. *P < .05; **P < .01; ***P < .005; comparison without an indication are nonsignificant. **(E)** Fungal genera showing significant differences in their relative abundance between duodenal and fecal samples in a DeSeq2 model correcting for sequencing depth.

The mycobiome (assessed through ITS2 sequencing) of the upper and lower GI tract differed significantly in its alpha- and beta-diversity. Fecal samples had a higher number of observed taxa, but lower evenness and thus a lower overall diversity as measured by the Shannon index (Figure 5A; *P* < .001). Further, the gastric and duodenal samples showed a similar composition (Figure 5C), and both differed significantly from the fecal samples when assessing relative abundance (Bray–Curtis index, Figure 5B; *P* = .006) and presence/absence (Jaccard index; Figure S9A, Supporting Information; *P* = .002). The ratio of Basidiomycota/Ascomycota was significantly higher in gastric and duodenal samples compared to fecal samples (Figure 5C; *P* = .003). Several members of *Saccharomycetales* were found in higher levels in the feces compared to the upper GI tract, including *Saccharomyces, Kasachstania, Debaryomyces, Wickerhamomyces*, and *Meyerozyma*, while *Humicola* is more abundant in the upper GI samples (Figure 5D). Further, these fungal genera remained significantly different in between fecal and duodenal samples in a multivariate analysis correcting for inflammatory status, sequencing run and total fungal read count in the combined dataset (Figure 5E), and when the ITS dataset was stratified by country of origin (Figure S11, Supporting Information). The same trends were also observed in a reduced sample set including only subjects with samples for all three GI tract locations (Figure S12, Supporting Information).

Our data thus clearly shows that the eukaryome differs in between different sites of the GI tract.

## Discussion

Eukaryotes are important members of the GI tract, contributing to the consumption of nutrients and vitamin production (Schei et al. 2017) and directly modulating both the bacterial microbiota composition (Morton et al. 2015, Zaiss et al. 2015, Audebert et al. 2016, Nieves-Ramirez et al. 2018, Stensvold and van der Giezen 2018) and the immune system (Underhill and Iliev 2014, Zaiss et al. 2015, McFarlane et al. 2017, Richard and Sokol 2019). While the field of parasitology is well-established, the diversity and ecology of human intestinal eukaryome and variability across individuals remains poorly studied. We describe the human eukaryome and mycobiome in a large cohort of children living in Africa without diarrhea or acute GI symptoms. We find a diverse community of eukaryotes, which does not seem to be strongly influenced by clinical factors. The results here are consistent with the emerging viewpoint that many eukaryotes present in the human GI tract may be minimally harmful, commensal or of potential benefit (Parfrey et al. 2014, Andersen and Stensvold 2016, Stensvold and van der Giezen 2018). Pathogenic protists such as *E. histolytica* and *Cryptosporidium* sp. are major sources of morbidity and mortality among children in resource poor settings (Turkeltaub et al. 2015), and have rightly garnered much research attention. These taxa are rare here, likely because this study excluded children with acute GI disease (Vonaesch et al. 2018). Further, many carriers of *E. histolytica* and *G. intestinalis* do not show (severe) symptoms and their virulence is determined by a complex interplay between parasite, host, and microbiota composition (Marie and Petri 2014). Last, it has also been shown that the eukaryotic microbiota composition is associated with the bacterial microbiota composition (Morton et al. 2015) and that the bacterial community has in turn an influence on *Entamoeba* virulence in the human GI tract. Indeed, there is increased awareness that not only bacteria, but the whole microbial community inhabiting the GI tract, including protists, helminths, fungi, archaea, and viruses play an important role in the overall ecosystem as well as in cross-talk with the host and regulation of virulence [reviewed in Hirt (2019), Rowan-Nash et al. (2019), and Ungaro et al. (2019)]. Integrated studies across all domains of life would, therefore, be increasingly needed to understand the real implication of the human microbiome in health and disease.

To our knowledge, this is the first description of the eukaryotic community comparing different GI compartments and the first sequencing-based study to assess the eukaryotes in the human stomach and small intestine. Few gastric and duodenal samples yielded microeukaryotic reads in the 18S datasets, so we cannot compare the community across the GI tract. However, similar taxa were observed in the upper GI tract and in feces with *Blastocystis* most prevalent. Our data reveals that there is a clear compartmentalization in the communities found along the GI tract for fungi. Fungal composition was very similar between the gastric and duodenal samples, and both are distinct from the fecal composition, similar to the bacterial community (Vonaesch et al. 2018, 2022). While this might be expected, seen the vastly different environments encountered at each site, this has so far never been reported. Earlier research described few acid-tolerant fungi like *Candida* and *Phialemonium* (von Rosenvinge et al. 2013) [reviewed in Hallen-Adams and Suhr (2017)] in the stomach and culture-based approaches identified *Candida* in the small intestine (Heyworth and Brown 1975, Minoli et al. 1981). In our study, we assessed the fungal composition of 196 duodenal samples from two different countries and detect a vast diversity of strains, most of which are probably transients. Overall, the two most frequently detected taxa were *H. grisea* (70% in Madagascar, 84% in CAR) and *M. restricta* (52% in Madagascar, 71% in CAR). *Humicola grisea* is likely an environmental transient rather than a true colonizer of the gut: It is because it is a well-known thermophilic, soil-dwelling fungus and was not part of the eukaryotic community described in earlier studies assessing the feces in urban industrialized countries (Suhr and Hallen-Adams 2015, Hallen-Adams and Suhr 2017). *Malassezia* and *Cladosporium*, the two other most prevalent taxa in the upper GI tract, which are also frequently found in the feces here and in urban industrialized countries (Nash et al. 2017, Auchtung et al. 2018), show similar prevalence in all three compartments, suggesting that they could be true colonizers but further experimental work is necessary (Hallen-Adams and Suhr 2017). *Malassezia restricta* is also known to be a natural member of the human skin microbiota (Vijaya Chandra et al. 2020). *Malassezia* has also been associated with IBD in a recent patient study and its direct implication has been validated in subsequent experiments in mice (Limon et al. 2019).

In line with previous results from urban industrialized countries (Stensvold et al. 2011b, Parfrey et al. 2014) our data reveals that the fecal eukaryome of African children is dominated by fungi and protists, especially *Blastocystis* and *Entamoeba*. The patterns that there are few taxa in any given individual and high variability across the population are perhaps surprising when evaluated in the framework of the bacterial microbiome. Indeed, the fecal prokaryome harbours overall more taxa and a much higher proportion are widely shared across individuals. However, these patterns have been previously observed in humans and other mammals (Parfrey et al. 2014, Bachmann et al. 2015). The communities observed in this cohort are more diverse than in industrialized countries both in terms of the broader taxonomic groups represented (e.g. *Parabasalids, Blastocystis*) and greater diversity within taxonomic groups. At the individual level this means there are more so called mixed colonizations, e.g. multiple subtypes and strains of *Blastocystis* and *Entamoeba* were detected in ∼50% of individuals that harbour these taxa. We did not detect *Chilomastix, Dientamoeba*, nor *Cryptosporidium* in our 18S datasets, despite the fact they are widespread in the human fecal eukaryome (Barratt et al. 2011, Roser et al. 2013, Turkeltaub et al. 2015, Jokelainen et al. 2017, Greigert et al. 2018). There is evidence that *D. fragilis* is more prevalent in westernized compared to traditional communities (Barratt et al. 2011), which could explain the absence of *D. fragilis* in our study. Another hypothesis is that low sequencing depth was insufficient to detect *D. fragilis* and *Chillomastix*, which are low prevalence in the microscopy-based analysis. Last, we identified three primer mismatches of the primers for Parabasalids (*Dientamoeba*) and for Fornicata (including *Chilomastix*), which could explain the missing reads. *Cryptosporidium* is mainly associated with diarrhoea (Checkley et al. 1998, Costa et al. 2011, Mekonnen et al. 2019), so likely not detected in our cohort due to the choice of children included in the study. In line with this hypothesis, *Cryptosporidium* was also not detected through microscopy.

Last, recent evidence suggests that there are age-dependent changes not only in the bacterial microbiota community but also in the eukaryotes. Indeed, two studies in the USA and Ireland showed *Blastocystis* to be less prevalent in children compared to adults (Scanlan et al. 2016, 2018). This contrasts with the high prevalence of protist in the cohort of children described here. This difference might be due to a very high exposure to contaminated drinking water in these areas (Habib et al. 2021, Vonaesch et al. 2021), favouring the colonization by these microorganisms in exposed children. This would be in line with a previous study in children from Mexico, showing a high prevalence of *Blastocystis* already in early life (Partida-Rodriguez et al. 2021). More work is needed to understand the early life dynamics of eukaryotes within the human GI tract.

The fecal mycobiome of African children resembles the mycobiome reported in westernized countries [reviewed in Richard and Sokol (2019)]: in most studies, *Candida* (particularly *Candida albicans*), *Saccharomyces* (particularly *S. cerevisiae*), *Penicillium, Aspergillus, Cryptococcus, Malassezia* (particularly *M. restricta*), *Cladosporium, Galactomyces, Debaryomyces*, and *Trichosporon*, were detected in decreasing prevalence. In addition to these species, we frequently detected *Humicola, Kazachstania*, and *Wickerhamomyces. Humicola grisea* is an environmental fungus (Wang et al. 2019). *Kazachstania humilis*, also called *Candida humilis* and *K. exigua* are normally found in fermented food (Garcia-Ortega et al. 2022). *Wickerhamomyces anomalus* also called *Saccharomyces anomalus* or *Pichia anomala* is often associated with spoilage or processing of food (Masneuf-Pomarede et al. 2015) and has also been found in the GI tract of insects (Cappelli et al. 2020). Very few eukaryotic taxa were present in the feces of at least 50% of children, including *H. grisea* and *M. restricta* in the ITS2 dataset. Additional studies on describing the mycobiome of the food and environment in conjunction with the gut mycobiome, including ideally also source-tracking and/or repeated sampling would be needed to determine where gut fungi originate, and which are true colonizers of the gut (Lavrinienko et al. 2021). Here and in other studies fungal diversity within a sample (alpha diversity) is relatively low, Ascomycota and Basidiomycota are the dominant phyla, and many of the same genera are found (Hoffmann et al. 2013, Chehoud et al. 2015, Richard et al. 2015, Nash et al. 2017, Richard and Sokol 2019). The Ascomycota/Basidiomycota ratio was previously associated with gut health and has been shown to be lowered in adults (Sokol et al. 2017) as well as a children (Chehoud et al. 2015) suffering of IBD. However, in our study set, there was no association between this ratio and either stunting, anemia, or intestinal inflammation. However, there was a clear difference in the ratio by country of origin with higher Basidiomycota levels in Madagascar compared to CAR.

Which of the eukaryotes truly colonize the human gut and which are only transient is a fundamental but unanswered question and one, i.e. increasingly debated for fungi (Hallen-Adams and Suhr 2017, Fiers et al. 2019). Recent evidence suggests that fungal colonization of the intestinal tract of healthy individuals is minimal (Auchtung et al. 2018), and raises the possibility that a small minority of the fungi detected in the gut mycobiome are residents of the gut (exemplified by *Candida albicans*; Fiers et al. 2019). More than two-thirds of all species reported in two previous studies were found only in a single sample, suggesting that they come from environmental sources (Suhr and Hallen-Adams 2015, Hallen-Adams and Suhr 2017). We hypothesized that true residents should be likely shared across several individuals, while food contaminants might be distributed less consistently. We thus tried to address this point by filtering the taxa according to their prevalence. Of note that however even commonly detected gut fungi, such as *Debaryomyces hansenii* or *Penicillium*, do not grow at 37°C and might thus not be true residents of the gastrointestinal tract (Auchtung et al. 2018). Other common fungi may be transients and originate from food (e.g. *Saccharomyces cerevisiae*) are plant pathogens (such as *Fusarium, Alternaria*, and *Botrytis*), fungal communities from other parts of the body (*Malassezia*), or from the environment (*Aspergillus*) [reviewed in Auchtung et al. (2018)]. It is impossible to determine without experimental evidence if fungi are transient or resident members of the microbiota (Fiers et al. 2019). Similarly, several of the microeukaryotes detected, such as the rotifer *Rotaria*, are common inhabitants of freshwater and therefore likely environmental contaminants. Even within known gut taxa we detect strains that are likely transient in humans and true residents of other mammals, such as *E. bovis*. These results reiterate the importance of critically examining the eukaryotic community rather than assuming all sequences obtained are members of the eukaryome.

Transient fungi might contribute to intestinal disturbances, especially if they produce mycotoxins (Smith et al. 2012); *F. equiseti, Penicillium, Fusarium*, and *Aspergillus* are among the mycotoxin producers detected here (Goswami et al. 2008, Munkvold 2017). *Fusarium equiseti* is a plant pathogen of cereals, field weeds, durian, goji berries, among others and is found in both, tropical and temperature regions (Goswami et al. 2008, Munkvold 2017). Further, colonization with *F. equiseti* has been associated with a vegetarian diet (Hallen-Adams and Suhr 2017). Interestingly, we found a consistent trend towards higher levels of *F. equiseti* in stunted children compared to healthy controls. This association might be due to a diet low in meat and high in plant matter consumed by stunted children (Vonaesch et al. 2021) and favouring the colonization by *F. equiseti* (Hallen-Adams and Suhr 2017). It is also tempting to speculate that this fungus is contributing to the pathophysiology underlying stunted growth, however, mycotoxin profiling as well as experimental data are needed to establish a causal relationship between these toxins and intestinal disturbances.

The diversity of protists and nematodes detected here resembles earlier reports from humans living in Sub-Saharan Africa with high prevalence of *Blastocystis, Entamoeba, Trichomonads*, and yeasts (Parfrey et al. 2014, Greigert et al. 2018). The catalogue of gut eukaryotes observed here is comparable to other parts of the world, albeit with higher diversity than typically found in Europe (Forsell et al. 2012, Scanlan et al. 2018, Lhotska et al. 2020). Nematodes (especially *Ascaris* and *Trichuris trichiura*) were mainly found in Madagascar and only rarely in the CAR, possibly related to deworming medicine or lifestyle choices. However, local hotspots of nematode colonization were previously reported from several places in Africa and are not associated with deworming campaigns (Moser et al. 2017, Schulz et al. 2018).

Diversity within common gut protists is high: diverse *Entamoeba* species colonize the intestines of humans and nonhuman primates (Stensvold et al. 2011b, Elsheikha et al. 2018, Stensvold 2019, Dos Santos Zanetti et al. 2021), and in this cohort we frequently detected *E. polecki, E. dispar/E.histolytica, E. hartmanni*, and *E. coli. Entamoeba bovis* is most often associated with ungulates and is a potential transient here. *Entamoeba histolytica*, a true pathogen, is detected less frequently and difficult to distinguish from *E. dispar* by 18S sequencing (a specific qPCR is needed to make the molecular distinction). The diversity of *Entamoeba* detected here is high, and half of individuals positive for *Entamoeba* harbor multiple species and/or subtypes.

We find a trend towards greater *Entamoeba* presence in nonstunted compared to stunted children. Two studies assessing a possible correlation between infection with specific pathogens and stunted growth are currently ongoing within the Afribiota project.

*Blastocystis* is the most common protist within this cohort and widespread in humans and other mammals. Overall, *Blastocystis* ST1, ST2, and ST3 are predominant here, as in other human populations (Stensvold et al. 2020, Forsell et al. 2012), and ST4, which is common in Europe is absent. The diversity of *Blastocystis* is similar between countries , though the prevalence of several strains (subclusters) differs between countries . In contrast to two previous studies in Europe (Flemish Gut Project and Twins UK) (Tito et al. 2019), more than half of the children included in our study showed concomitant presence of several *Blastocystis* subtypes in their feces. Our results suggest that different *Blastocystis* subtypes and *Entamoeba* species often coexist. This confirms a recent study in Cameroon in which it was shown that some subtypes of *Blastocytis* and *Entamoeba* species show a positive correlation in their occurrence (Even et al. 2021).

One surprising finding that emerges from our study is the low correlation between the eukaryome and clinical variables. In previous studies, particular fungal taxa were associated with increased inflammation in the context of IBD and/or colorectal cancer (Ye et al. 2006). In industrialized countries lower prevalence of *Blastocystis* has been reported in patients with inflammatory diseases including colorectal cancer or Crohn’s disease compared to control individuals (Beghini et al. 2017, Tito et al. 2019). Indeed, these microaerobic protists thrive in low oxygen levels, while higher oxygen levels are a hallmark of gut inflammation, and *Blastocystis* could, therefore, be a marker of a ‘healthy gut environment’, meaning a gut environment that remains largely anaerobic (Audebert et al. 2016, Stensvold et al. 2020). *Entamoeba* is also reported to be inversely correlated with inflammatory diseases (Morton et al. 2015). Here we see trends of lower prevalence for *E. coli* in stunted children compared to nonstunted controls, though the association was not significant after correcting for multiple testing. While these results might be confounded by the fact that we had only limited sequencing depth and a very high variability of taxa for fungi, likely decreasing statistical power, our results suggest the eukaryotic community, at least in our two study sites, is not dramatically reorganized by the clinical variables measured. More research is needed to better elucidate the role of eukaryotes to the pathophysiology associated with a dysbiotic microbiota.

Overall, our results from two sites in Sub-Saharan Africa show little correlation between stunting and the eukaryome, or individual eukaryote prevalence. This could suggest that eukaryotes are less directly involved in the pathophysiology of chronic childhood undernutrition, as suggested also in a targeted analysis of the parasites in the Madagascar study site of Afribiota (Habib et al. 2021). Our results could also be a reflection that eukaryotes are less influenced by the altered gut environment compared to bacteria (Vonaesch et al. 2018). Further studies are, however, needed to corroborate this point and exclude any technical bias or site-specific effects. Earlier reports showing a direct role of helminths in undernutrition led to the recommendation of WHO for systematic antihelminthic treatments. However, our observation is consistent with a recent meta-analysis on 80 studies showing that helminths are not directly involved in undernutrition (Raj et al. 2022). In line with this observation, a recent meta-analysis found very little impact of antihelminthic treatments on stunting or development (Taylor-Robinson et al. 2019). However, the cross-sectional design of our study does not allow to capture events that initiate pathophysiology of stunting and therefore, cannot rule out a role for eukaryotes in leading to long term undernutrition. Further, as many children harbor several eukaryotes at the same time, it is possible that effects of individual taxa are shielded because, e.g. different eukaryotes might have opposite effects on the immune system that cancel each other out.

This work provides a foundation for future studies assessing the eukaryome in disease contexts and longitudinal studies on the establishment and role of the human eukaryome. Future studies should also assess for interactions between prokaryotes and eukaryotes in the gut microbiome to reveal cross-kingdom community structures and dynamics potentially influencing gut homeostasis and disease and assess how these interactions are shaped by diet and subsistence, as previously shown to play a role in a study on nonhuman primates (Sharma et al. 2022) as well as in several studies in humans (Morton et al. 2015, Rowan-Nash et al. 2019).

One strength of our study lies in the fact that we combine the analysis of the 18S rRNA gene for the overall eukaryome with and without an additional primer blocking amplification of human DNA and the internally transcribed spacer (ITS) gene for targeted analyses of the mycobiome and that we confirm presence of given eukaryotes on a subset of samples using microscopy. We show that the use of a mammalian blocking primer altered the observed community structure by detecting higher diversity within a sample, especially of protists and microeukaroytes detected from a higher proportion of samples. This shows a limitation of amplicon-based profiling calls for standardized primers and protocols to allow for comparisons between different studies and locations. Combining the ITS and 18S rRNA gene approaches we were able to show that fungal and microeukaryotic diversity in the gut of African children are only marginally correlated with clinical factors, yet strongly shaped by geographic location, most likely through diet and other environmental exposures.

Our study has a few limitations: as a single sample was taken from each child and as we included only children aged 2–5 years. Therefore, the study does not allow assessing for dynamic changes nor does it allow to make any assumptions about early life succession. Further, storage protocols slightly differed across the two sites and DNA was extracted with the same kit but in two different geographic locations and by different experimenters. Thus, we might have overestimated geographic differences. The overall impact of geography on the microeukaryome was, however, confirmed on a subset of samples using microscopy. Further, seen that we only included two study sites in our project, the results about the overall diversity of eukaryotes might be specific to our study context and might not apply to other LIMC settings. Further, since the implementation of our study, new amplicon-based sequencing methods have been developed, allowing for the detection of a broader range of eukaryotes within the human microbiome (Popovic et al. 2018). Last, PCR based approaches used for the taxonomic profiling of a given microbiota are at best semiquantitative in nature and typically do not integrate various taxonomic groups. This hurdle can somehow be overcome by metatranscriptomic approaches, which can provide a more global, unbiased and quantitative approach, as exemplified in a recent publication on idiopathic chronic diarrhea in macaques (Westreich et al. 2019). Thus, as any sequencing-based study, the data presented here is likely not representative of the true diversity of eukaryotes found in the feces of these children.

Nevertheless, by comparing the eukaryome of almost 1000 children of whom roughly half showed fecal read counts for 18S rRNA gene and/or the ITS2 region in two different geographic locations of Sub-Saharan Africa, our data contributes valuable insights about the human eukaryome and sets the stage for more targeted analyses of eukaryome dynamics and of the role of the eukraoyme in health and disease.

In conclusion, our study clearly shows that African children harbour a specific eukaryome, which is compartmentalized along different sites of the GI tract and is strongly influenced by country of residence.

## Group authorship Afribiota investigators

AFRIBIOTA Investigators (Group authorship in alphabetical order):

Laurence Barbot-Trystram, Hôpital Pitié-Salpêtrière, Paris, France.

Robert Barouki, Hôpital Necker- Enfants maladies, Paris, France.

Alexandra Bastaraud, Institut Pasteur de Madagascar, Antananarivo, Madagascar.

Jean-Marc Collard, Institut Pasteur de Madagascar, Antananarivo, Madagascar.

Maria Doria, Institut Pasteur, Paris, France.

Darragh Duffy, Institut Pasteur, Paris, France.

B. Brett Finlay, University of British Columbia, Vancouver, Canada.

Serge Ghislain Djorie, Institut Pasteur de Bangui, Bangui, Central African Republic.

Tamara Giles-Vernick, Institut Pasteur, Paris, France.

Milena Hasan, Institut Pasteur, Paris, France.

Bolmbaye Privat Godje, Complexe Pédiatrique de Bangui, Bangui, Central African Republic.

Jean-Chrysostome Gody, Complexe Pédiatrique de Bangui, Bangui, Central African Republic.

Francis Allen Hunald, Service de Chirurgie pédiatrique, Centre Hospitalier Universitaire Joseph Ravoahangy Andrianavalona (CHU-JRA), Antananarivo, Madagascar.

Nathalie Kapel, Hôpital Pitié-Salpêtrière, Paris, France.

Jean-Pierre Lombart, Institut Pasteur de Bangui, Bangui, Central African Republic.

Alexandre Manirakiza, Institut Pasteur de Bangui, Bangui, Central African Republic.

Synthia Nazita Nigatoloum, Complexe Pédiatrique de Bangui, Bangui, Central African Republic.

Laura Wegener Parfrey, University of British Columbia, Vancouver, Canada.

Lisette Raharimalala, Centre de Santé Materno-Infantile, Tsaralalana, Antananarivo, Madagascar.

Maheninasy Rakotondrainipiana, Institut Pasteur de Madagascar, Antananarivo, Madagascar.

Rindra Randremanana, Institut Pasteur de Madagascar, Antananarivo, Madagascar.

Harifetra Mamy Richard Randriamizao, Centre Hospitalier Universitaire Joseph Ravoahangy Andrianavalona (CHU-JRA), Antananarivo, Madagascar.

Frédérique Randrianirina, Institut Pasteur de Madagascar, Antananarivo, Madagascar.

Annick Robinson, Centre Hospitalier Universitaire Mère Enfant de Tsaralalana, Antananarivo, Madagascar.

Pierre-Alain Rubbo, Institut Pasteur de Bangui, Bangui, République Centrafricaine.

Philippe Sansonetti, Institut Pasteur, Paris, France.

Laura Schaeffer, Institut Pasteur, Paris, France.

Ionela Gouandjika-Vassilache, Institut Pasteur de Bangui, Bangui, République Centrafricaine.

Pascale Vonaesch, Institut Pasteur, Paris, France.

Sonia Sandrine Vondo, Complexe Pédiatrique de Bangui, Bangui, Central African Republic.

Inès Vigan-Womas, Institut Pasteur de Madagascar, Antananarivo, Madagascar.

## Supplementary Material

uqad033_Supplemental_Files

## Notes

The Afribiota Investigators are listed in the acknowledgements.

Reviewer Name(s): Magali Chabé, Heather Hallen-Adams, Pauline Scanlan
