# Supplementary material for: The eukaryome of African children is influenced by geographic location, gut biogeography, and nutritional status
Source: Microlife. 2023 Jul 20;4:uqad033. doi: 10.1093/femsml/uqad033 (PMC10481997; doi:10.1093/femsml/uqad033)
Supplement: uqad033_Supplemental_Files [file uqad033_supplemental_files.zip › Supplementary Table S3.docx]

***Table S3: Overall characteristics of the children included***

| **Dataset** | ITS | | 18S no block | | 18S w/block^$^ | |
| --- | --- | --- | --- | --- | --- | --- |
|  | ***N=315**** | | ***N=463**** | | ***N=241**** | |
| **Country** | ***M*** | ***CAR*** | ***M*** | ***CAR*** | ***M*** | ***CAR*** |
|  | ***211*** | ***104*** | ***261*** | ***202*** | ***150*** | ***91*** |
| **Sex** |  |  |  |  |  |  |
| Female | 114 | 49 | 138 | 104 | 78 | 49 |
| Male | 97 | 55 | 123 | 98 | 72 | 42 |
| **Age** |  |  |  |  |  |  |
| 2-3 years | 65 | 40 | 87 | 94 | 52 | 31 |
| 3-4 years | 65 | 39 | 89 | 58 | 48 | 35 |
| 4-5 years | 81 | 25 | 85 | 50 | 50 | 25 |
| **Nutritional status** |  |  |  |  |  |  |
| Non-stunted | 111 | 50 | 132 | 95 | 61 | 54 |
| Stunted | 100 | 54 | 129 | 107 | 89 | 37 |
| **Fecal calprotectin** |  |  |  |  |  |  |
| Normal | 125 | 84 | 142 | 156 | 80 | 65 |
| High | 70 | 16 | 94 | 41 | 53 | 24 |
| Not measured | 16 | 4 | 25 | 5 | 17 | 2 |
| **Fecal α-antitrypsin** |  |  |  |  |  |  |
| Normal | 156 | 89 | 186 | 177 | 107 | 83 |
| High | 37 | 10 | 49 | 18 | 26 | 5 |
| Not measured | 18 | 5 | 26 | 7 | 17 | 3 |
| **Anemia^#^** |  |  |  |  |  |  |
| No | 158 | 50 | 195 | 90 | 106 | 36 |
| Yes | 47 | 43 | 61 | 86 | 38 | 37 |
| Not measured | 6 | 12 | 5 | 26 | 6 | 18 |

* subjects with > 5’000 reads in their fecal samples

# hemoglobin values were adjusted for altitude^22,23^ and anemia was defined as less than 110 g/l, according to WHO criteria^20,24^

^$^ eukaryotes unassigned beyond kingdom levels were excluded
