# Supplementary material for: The eukaryome of African children is influenced by geographic location, gut biogeography, and nutritional status
Source: Microlife. 2023 Jul 20;4:uqad033. doi: 10.1093/femsml/uqad033 (PMC10481997; doi:10.1093/femsml/uqad033)
Supplement: uqad033_Supplemental_Files [file uqad033_supplemental_files.zip › Supplement_Afribiota18S_210723.pdf]

Supplementary data for

"The eukaryome of African children is influenced by geographic location, gut biogeography, and nutritional status"

Pascale Vonaesch[1, 2, #], Vincent Billy[3], Allison E. Mann[3, 4], Evan Morien[3], Azimdine Habib[5], Jean-Marc Collard[5], Michel Dédé[6], Nathalie Kapel[7], Philippe J. Sansonetti[1, 8, #] & Laura Wegener Parfrey[3, #] for the Afribiota Investigators[$]

[$] the Afribiota Investigators are listed in the acknowledgements.

1: Unité de Pathogénie Microbienne Moléculaire, Institut Pasteur, 25-28 Rue du Dr Roux, 75015 Paris, France

2: current address: Department of Fundamental Microbiology, University of Lausanne, Campus UNIL-Sorge, 1015 Lausanne, Switzerland

3: Departments of Botany and Zoology, and Biodiversity Research Centre, University of British Columbia, 3200-6270 University Boulevard, V6T1Z4 Vancouver, Canada

4: current address: Department of Biological Sciences, Clemson University, 132 Long Hall, Clemson, SC 29631, USA

5: Unité de Bactériologie Expérimentale, Institut Pasteur de Madagascar, BP1274 Ambatofotsikely Avaradoha 101 Antananarivo, Madagascar

6: Laboratoire d'Analyse médicale, Institut Pasteur de Bangui, Avenue De Independence Bangui, 923 Central African Republic

7: Laboratoire de Coprologie Fonctionnelle, Assistance Publique- Hôpitaux de Paris, Hôpital Pitié-Salpêtrière, 47-83 Bd de l'Hôpital, 75013 Paris, France

8: current address: The Center for Microbes, Development and Health, Institut Pasteur of Shanghai and Chinese Academy of Sciences, 411 Hefei Rd, Huangpu, Shanghai, China

# Corresponding authors: Laura Wegener Parfrey lwparfrey@botany.ubc.ca; Pascale Vonaesch pascale.vonaesch@unil.ch; Philippe Sansonetti philippe.sansonetti@pasteur.fr

This supplement contains:

Supplementary Figures S1-S13

Supplementary File 1

Supplementary Tables S1-S19

Supplementary Figures

A

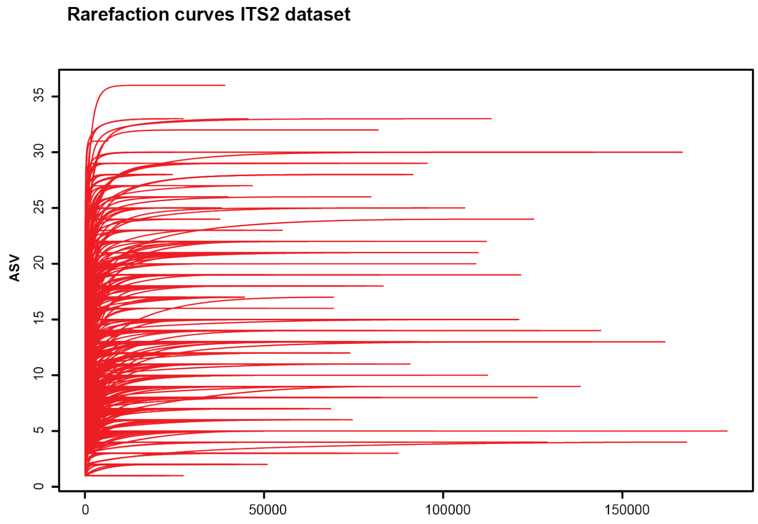

B

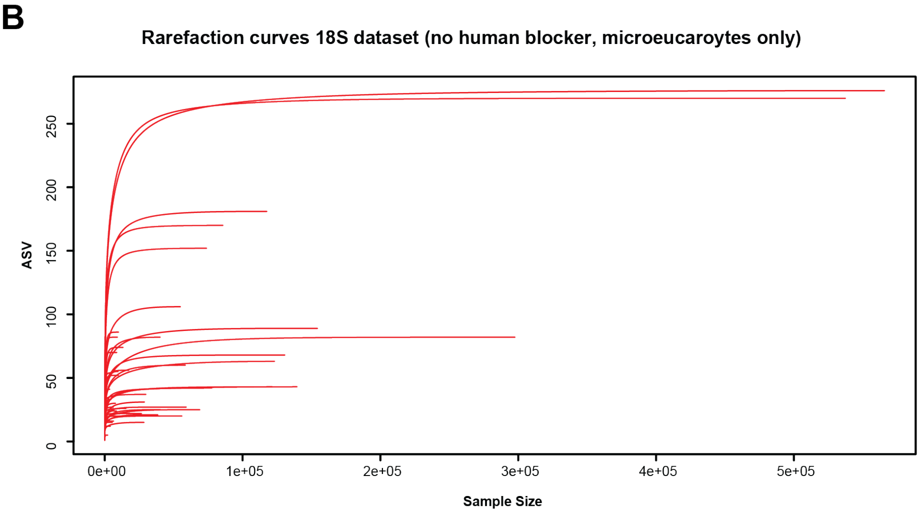

C

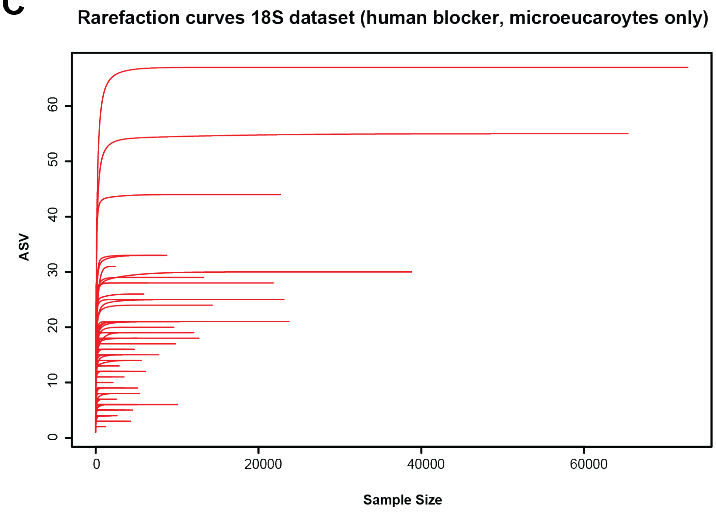

Supplementary Figure S1: Rarefaction curves of the three datasets analyzed: A) *ITS2 dataset* ***B)*** *18S dataset not using a mammalian blocking primer and* ***C)*** *subset using the mammalian blocking primer, filtering for vertebrate and plant sequences and keeping only the microeukaryotic reads.*

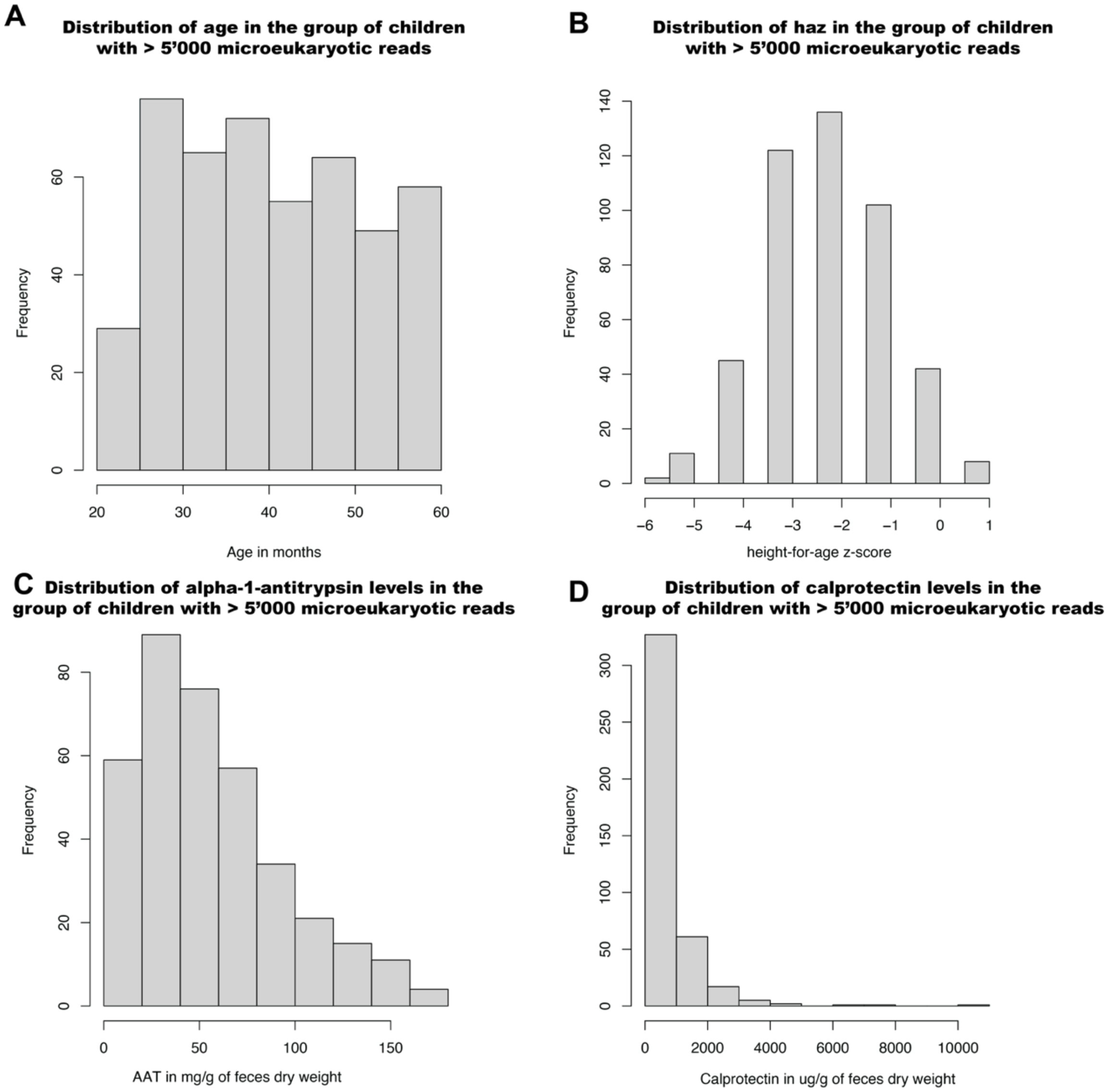

Supplementary Figure S2: Distribution of the main continuous, clinical variables analysed within the 18S dataset using no blocking primer: ***A)*** *Age in months* ***B)*** *height-for-age z-score* ***C)*** *alpha-1-antitrypsin (AAT) levels and* ***D)*** *calprotectin levels.*

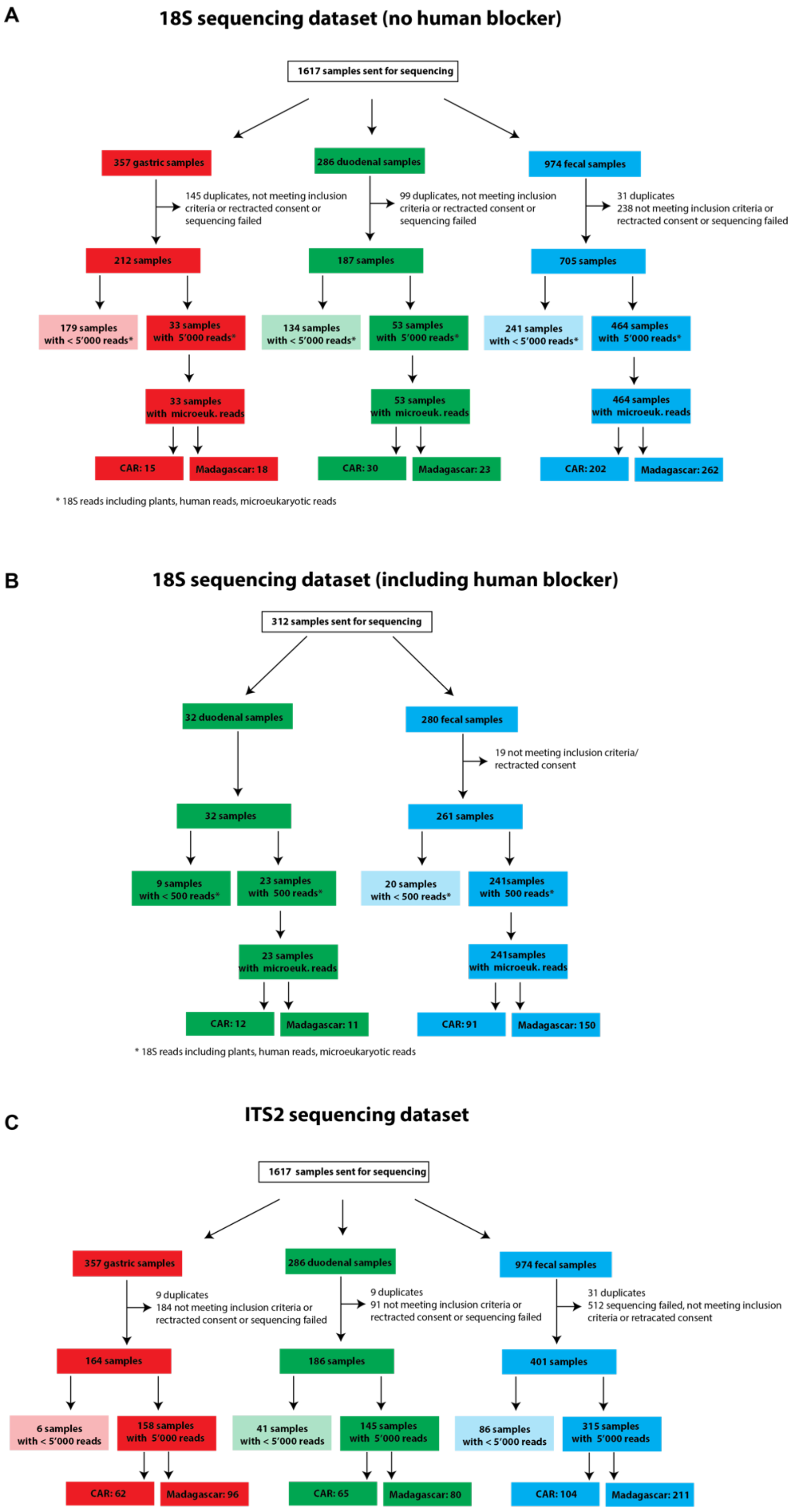

Supplementary Figure S3: Samples included in the final analysis. A) *18S dataset not using a mammalian blocking primer and* ***B)*** *subset using the mammalian blocking primer and* ***C)*** *ITS2 dataset.*

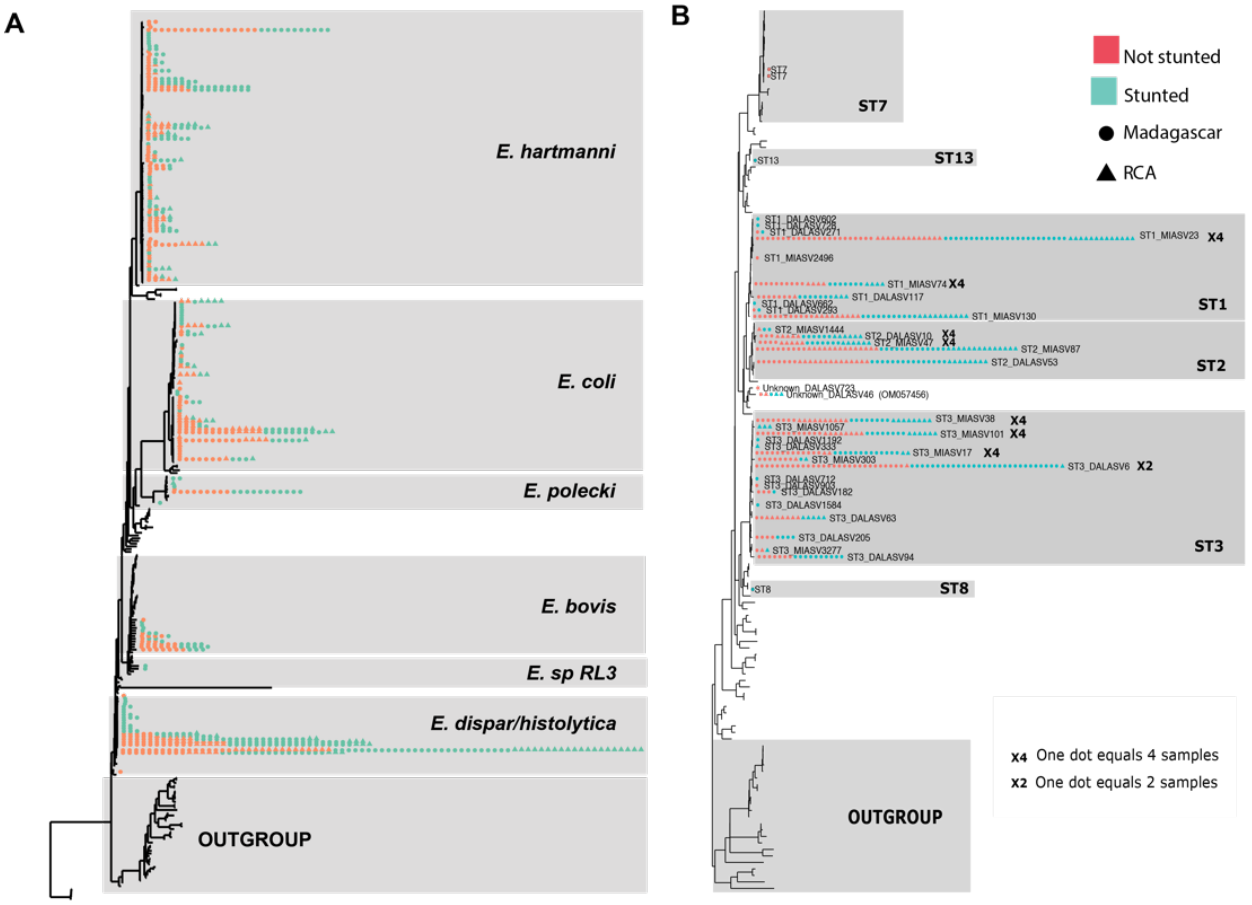

Supplementary Figure S4: Phylogenetic relationship of Blastocystis Subtypes and Entamoeba species. (A) *Placement tree of Blastocystis ASV and* ***(B)*** *the Entamoeba ASVs detected in the current dataset. Colors indicate the stunting status of children: red sequences were retrieved from non-stunted and blue sequences from stunted children. Shapes indicate the country of origin of children with circle representing samples from Madagascar and triangles samples from the Central African Republic (CAR).*

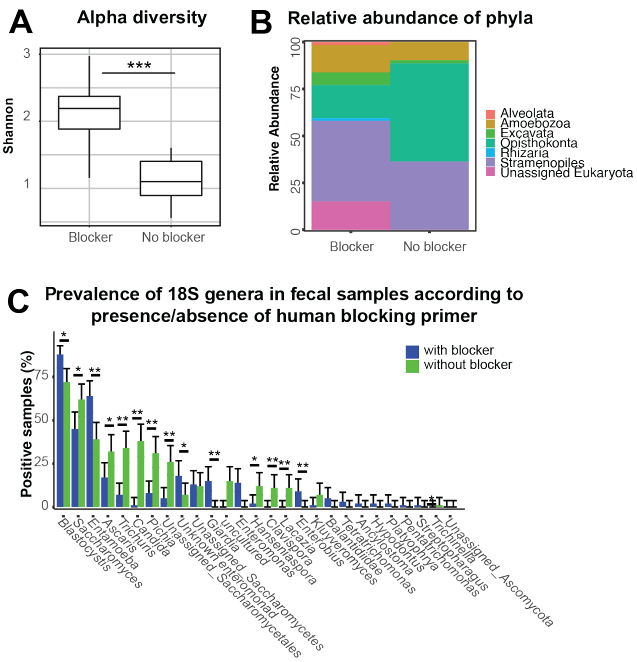

Supplementary Figure S5: Effect of the addition of a mammalian blocking primer to the microeukaryotic community structure retrieved. *Only samples sequenced with and without the blocking primer were included in the analysis (n=100). Comparison of the α-diversity of the dataset sequenced with and without a mammalian blocker using the* ***A)*** *Shannon index* ***B)*** *Chao1 index and* ***C)*** *Inversed Simpson index. Groups were compared using the Wilcoxon rank sum test.* ***D)*** *Comparison between the percentage of samples harboring sequences of the given eukaryotic genus. Groups were compared using the Pearson Chi2 test and Benjamini-Hochberg correction for multiple testing.* ***E)*** *Relative abundance of samples at class level by country of origin. *p<0.05; **p<0.01; ***p<0.005; comparison without an indication are non-significant.*

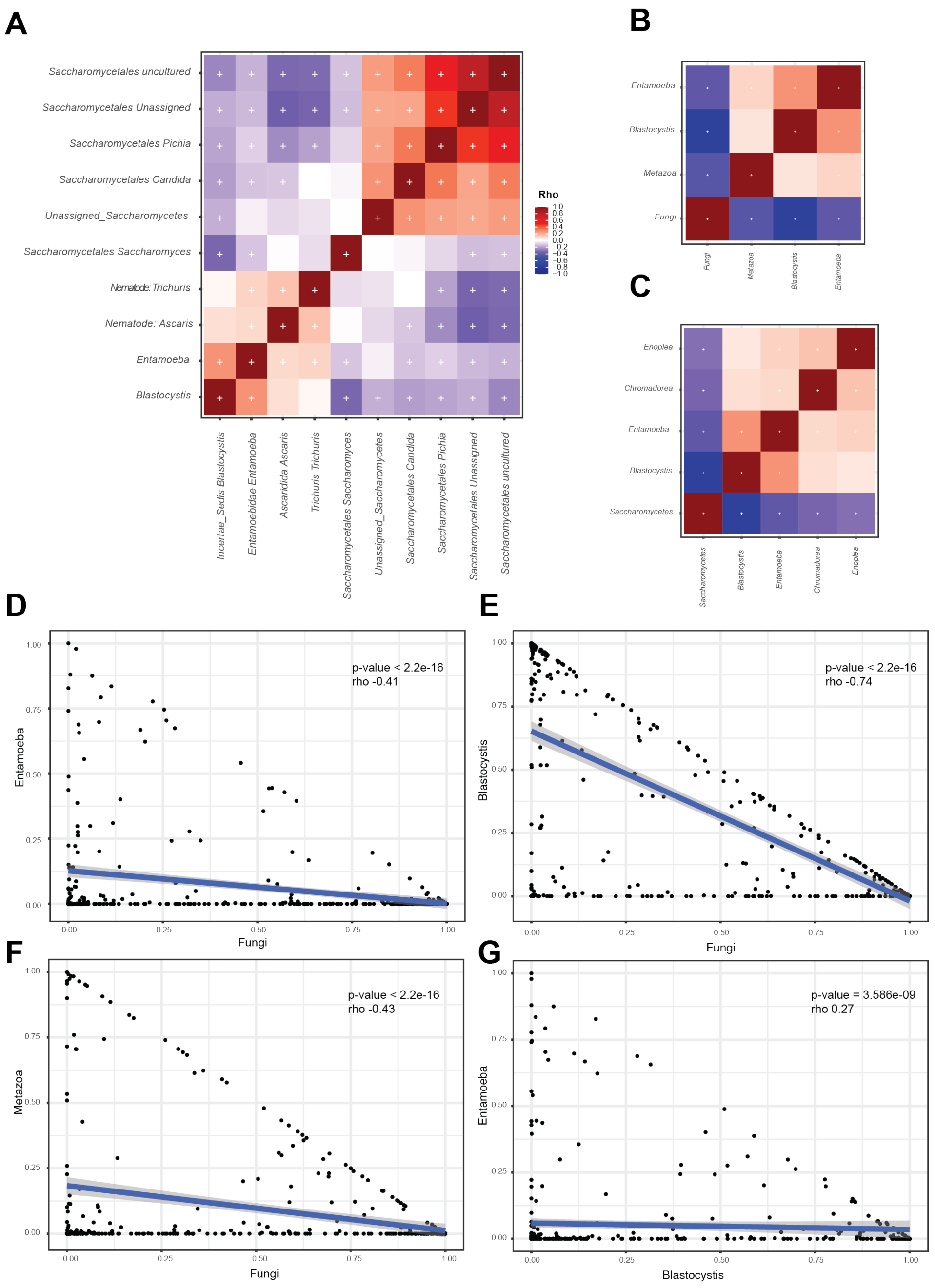

Supplementary Figure S6: Co-occurrence of microeukaryotes. *A) Heatmap of the Spearman correlation of the different genera in the 18S dataset using a mammalian blocker.* ***B) & C)*** *Heatmaps of the Spearman correlation of the taxa at different ranks in the 18S dataset not using a mammalian blocker.* ***D)- G)*** *Spearman correlation of given combinations of different microeukaryotic orders (rank3) showing a significant co-occurrence or co-exclusion. All datasets were restricted on taxa present in at least 10% of the samples with a relative abundance of at least 0.1%. The heatmap color code (rho) for panels A, C and F is given in panel A and comparisons marked with a + show a*

*significant association (p<0.05). Numbers in panel B give a numeric value of rho. * p<0.05; ** p<0.01; *** p<0.005;.*

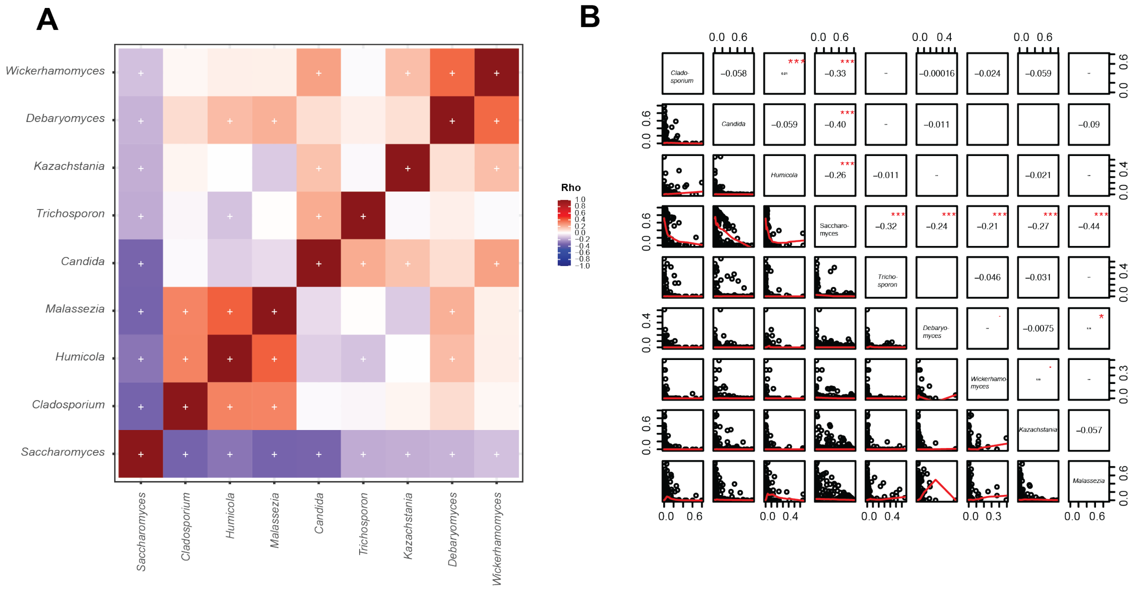

Supplementary Figure S7: Co-occurrence of different fungal genera. A) *Heatmap and* ***B)*** *scatter plot of the Spearman correlation of the different genera in the dataset restricted on taxa present in at least 10% of the samples with a relative abundance of 0.1%. The heatmap color code (rho) is given in panel A and comparisons marked with a + show a significant association (p<0.05). Numbers in panel B give a numeric value of rho. * p<0.05; ** p<0.01; *** p<0.005; comparison without an indication are non-significant.*

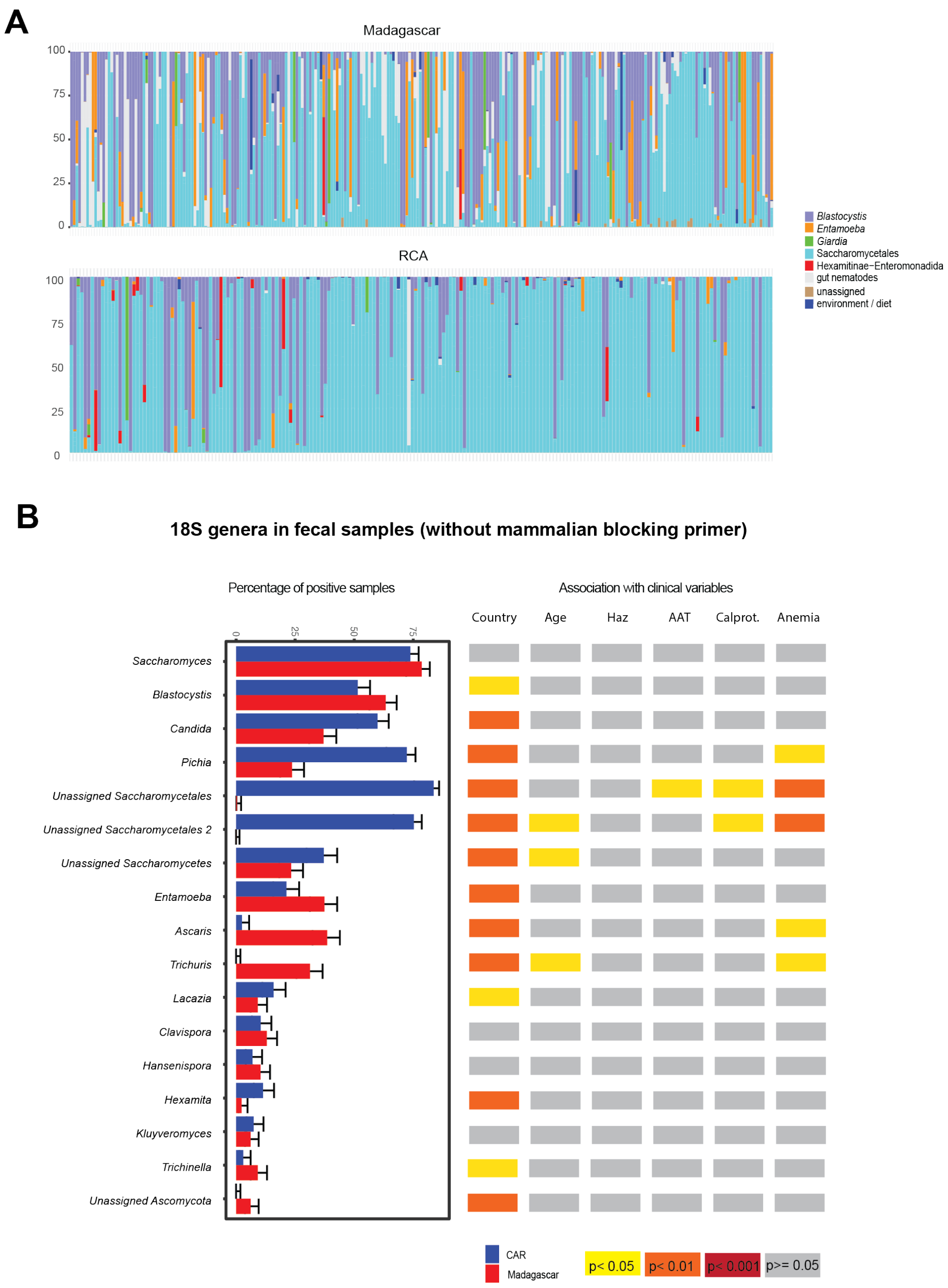

Supplementary Figure S8: Distribution of 18S genera in the dataset using no mammalian blocker. ***A)*** *Relative abundance of individual taxa/ taxa groups in the Afribiota samples as revealed by 18S sequencing* ***B)*** *Differences in the fecal microeukaryome in relation to geographic location and different clinical outcomes. Samples were considered to be positive for a given genus if they had at least a single sequence relating to this genus. Groups were compared using the Pearson Chi2 test and Benjamini-Hochberg correction for multiple testing. The color code illustrating the degree of significance of the association is given on the bottom of the figure*

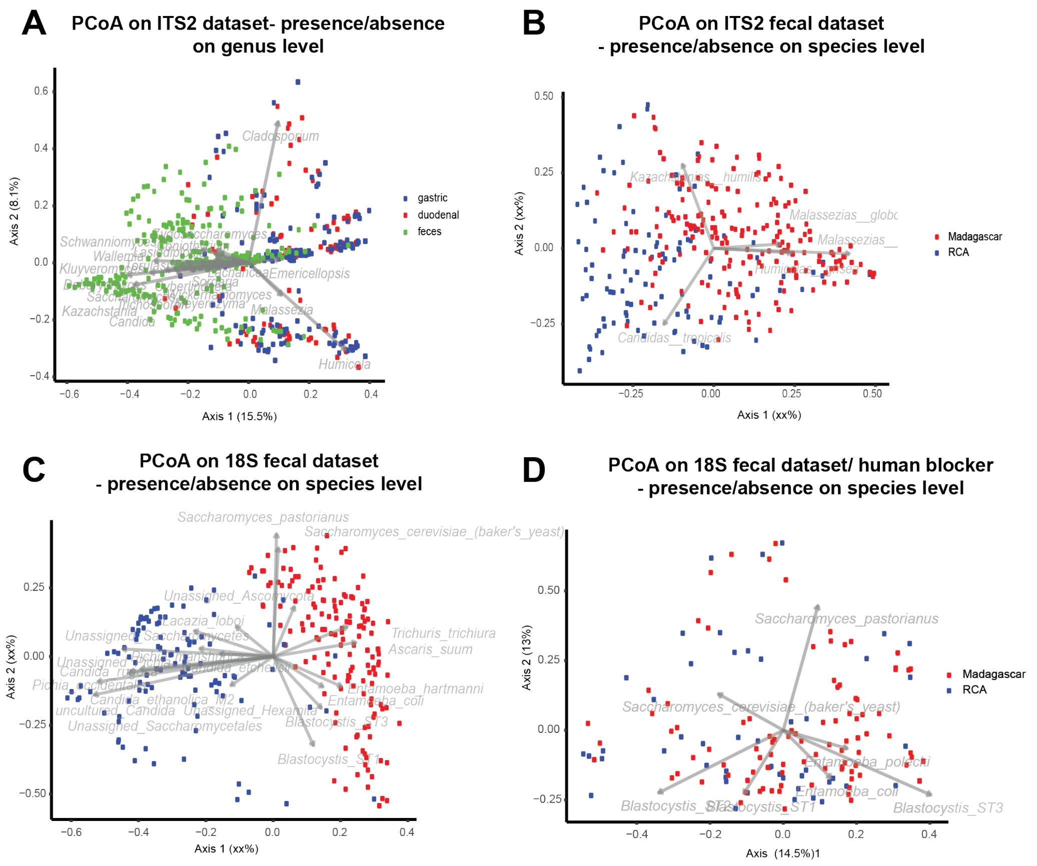

Supplementary Figure S9: Composition of the ITS2 and 18S dataset based on presence and absence of given taxa. A) *PCoA plot of the ITS2 dataset rarefied to 5,000 fungal sequences based on the Jaccard index indicating the samples by sampling location. Gastric samples (n=148) are indicated in blue, duodenal samples (n=132) in red and fecal samples (n=299) in green.* ***B)*** *PCoA plot of the ITS2 dataset rarefied to 5,000 fungal sequences based on the Jaccard index indicating the samples by country of origin. Samples from the Central African Republic (CAR, n=100) are indicated in blue, samples from Madagascar (n=202) in red.* ***C)*** *PCoA plot of the 18S dataset not using a mammalian blocker rarefied to 1,000 microeukaryotic sequences based on the Jaccard index indicating the samples by country of origin. Samples from the Central African Republic (CAR, n=108) are indicated in blue, samples from Madagascar (n=150) in red.* ***D)*** *PCoA plot of the 18S dataset rarefied to 1,000 microeukaryotic sequences based on the Jaccard index indicating the samples by country of origin. Samples from the Central African Republic (CAR, n=54) are indicated in blue, samples from Madagascar (n=99) in red.*

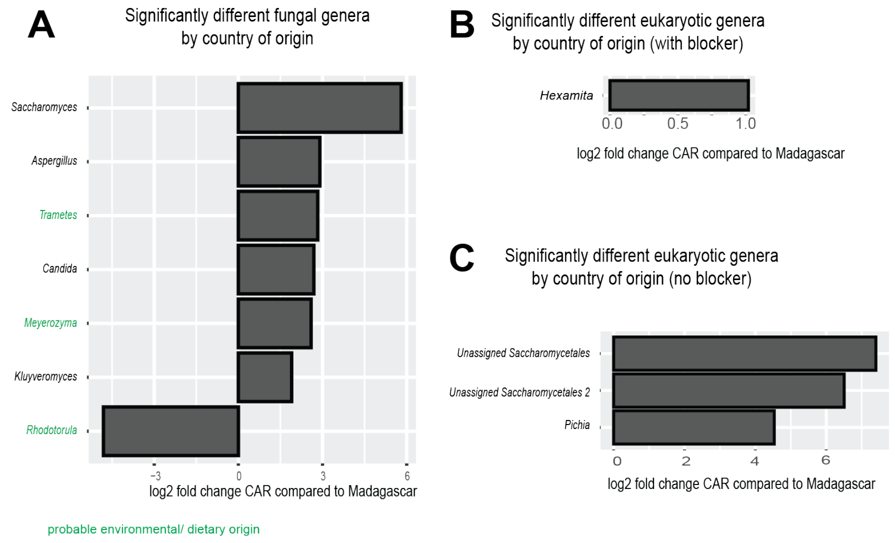

Supplementary Figure S10: Significantly different microeukaryotic taxa by country of origin. A) *Fungal genera (ITS2 dataset)* ***B)*** *microeukaryotic genera (revealed by 18S sequencing with a mammalian blocker) and* ***C)*** *microeukaryotic genera (revealed by 18S sequencing without a mammalian blocker) showing significant differences in their relative abundance in the two study countries in a DeSeq2 model correcting for sequencing depth, age and intestinal inflammation.*

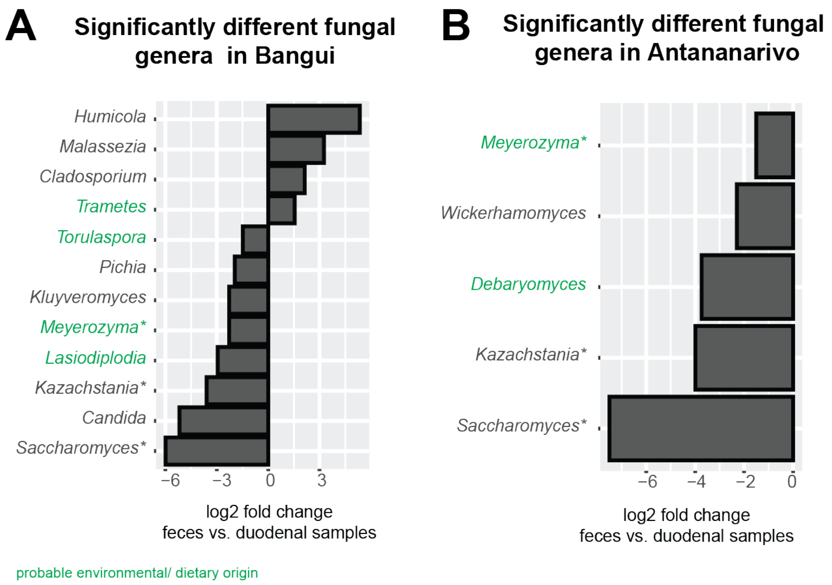

Supplementary Figure S11: Significantly different fungal taxa by sampling location. *Fungal genera showing significant differences in their relative abundance between duodenal and fecal samples in a DeSeq2 model correcting for sequencing depth in **A)** Bangui, Central African Republic and **B)** Madagascar.*

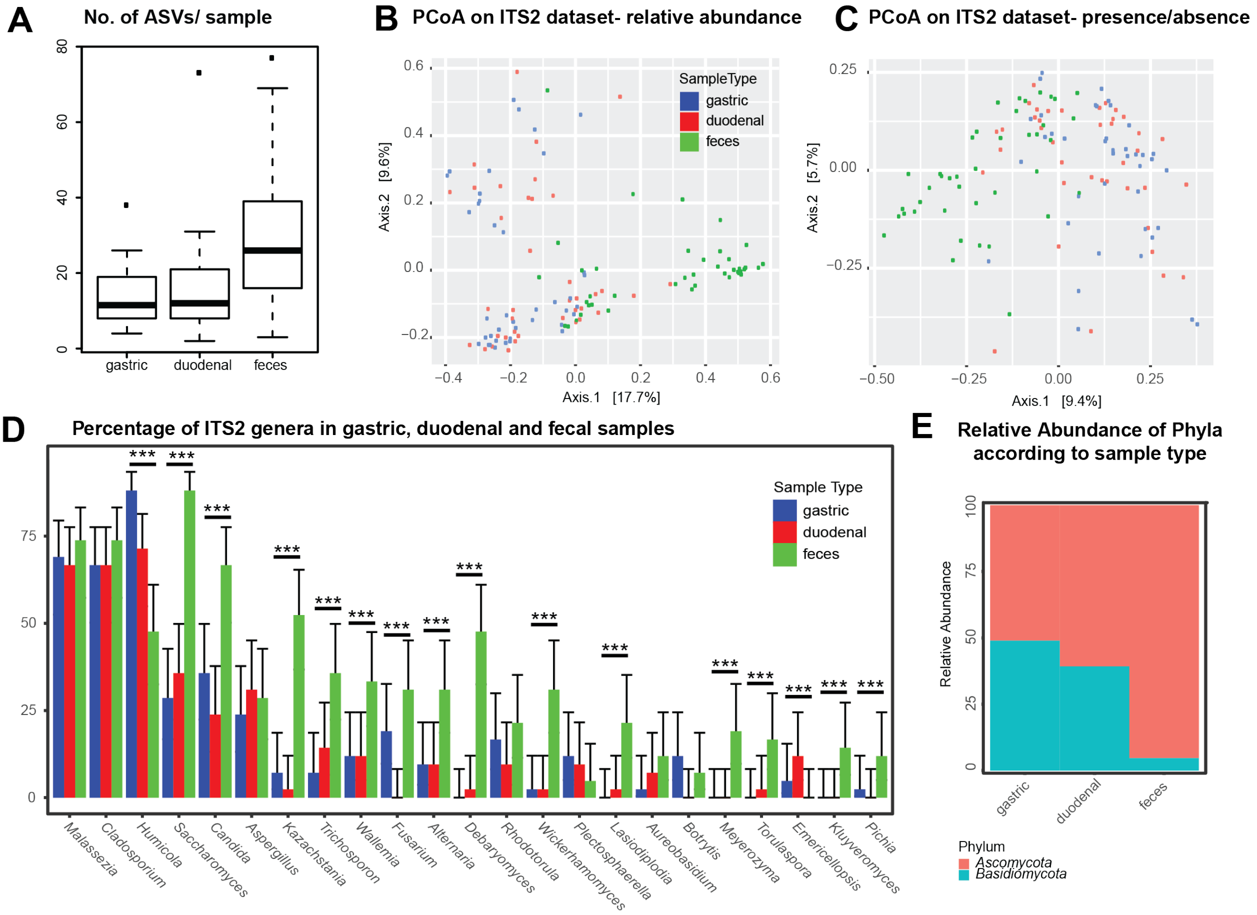

Supplementary Figure S12: Differences in the mycobiome based on sampling location on a reduced dataset comprising only subjects with samples for all three sampling locations. A) *Number of ITS2-ASVs retrieved in the non-rarefied dataset consisting of gastric (n=28), duodenal (n=28) and fecal (n=28) samples with more than 5,000 fungal sequences.* ***B)*** *PCoA plot based on the normalized Bray-Curtis dissimilarity index (log10) of the dataset iteratively rarefied to 5,000 fungal sequences. Gastric samples (n=34) are colored in blue, duodenal samples (n=30) in red and fecal samples (n= 30) in green.* ***C)*** *PCoA plot based on the Jaccard index of the presence-absence dataset iteratively rarefied to 5,000 fungal sequences. Gastric samples are colored in blue, duodenal samples in red and fecal samples in green.* ***D)*** *Comparison between the percentage of samples harboring sequences of the given fungal genus. Groups were compared using the Pearson Chi2 test and Benjamini-Hochberg correction for multiple testing * p<0.05; ** p<0.01; *** p<0.005; comparison without an*

indication are non-significant. ***E)*** *Average relative abundance of fungal phyla according to sampling location on the non-rarefied dataset.*

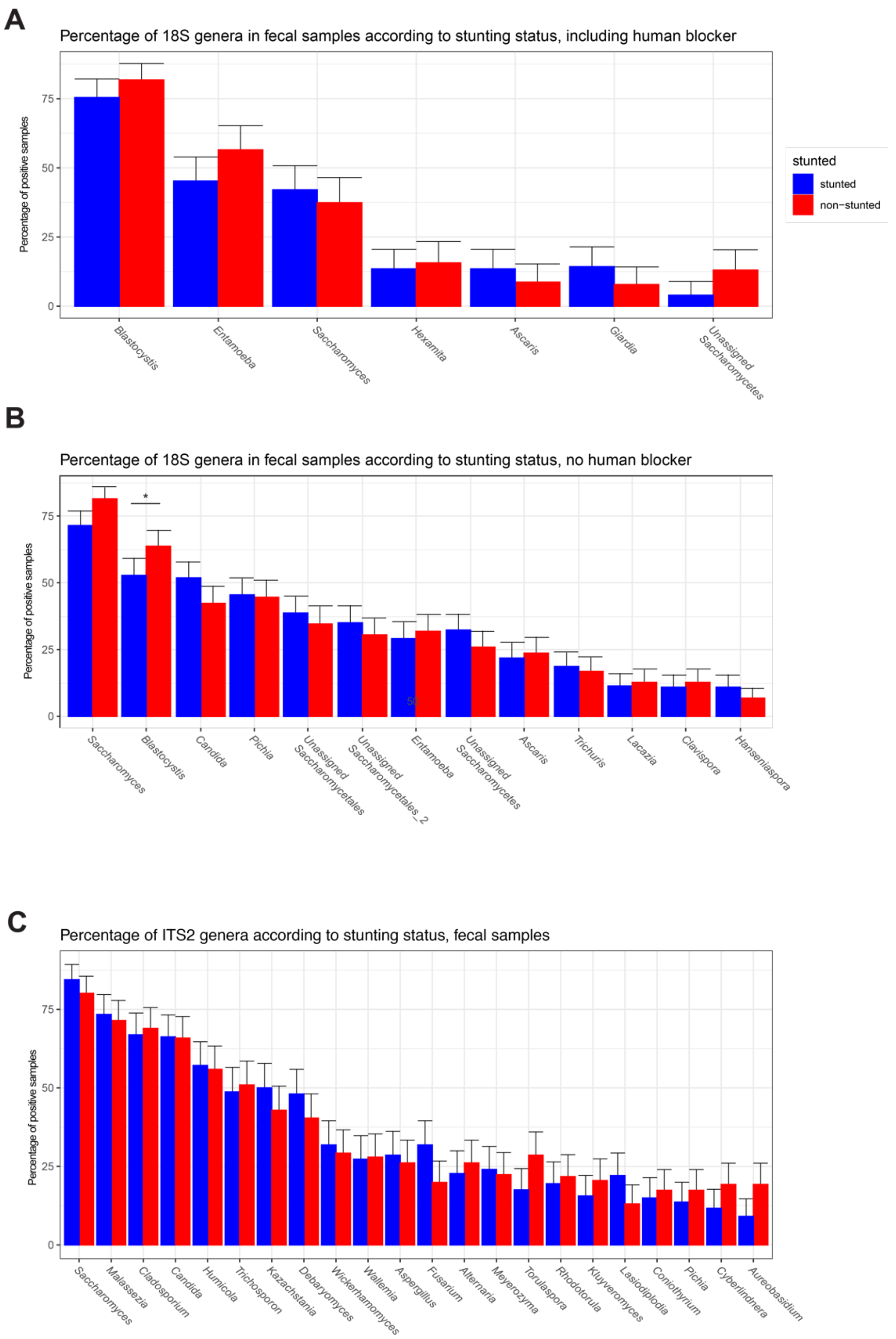

Supplementary Figure S13: Prevalence of microeukaryotic taxa by stunting status. *Comparison between the percentage of samples harboring sequences of the given eukaryotic genus based on stunting status.* ***A)*** *dataset without a human blocking primer,* ***B)*** *reduced dataset with a human blocking primer and* ***C)*** *ITS2 dataset. Groups were compared using the Pearson Chi2 test and Benjamini-Hochberg correction for multiple testing. * p<0.05; ** p<0.01; *** p<0.005; comparison without an indication are non-significant.*

Supplementary Files

Supplementary File 1: STORMS Microbiome checklist

Supplementary Tables

Supplementary Table S1: Summary of changes to the original taxonomy table (.xls file) *Changes are based on based on the phylogenetic trees, the correction of misannotated taxonomy by BLAST against NCBI.*

Supplementary Table S2: Backbone trees for the phylogenetic placement of sequences (.xls file)

Supplementary Table S3: Overall characteristics of the children included in the analysis

Supplementary Table S4: Co-colonization of different Blastocystis subtypes in the fecal samples. Presence was fixed as a minimum of 5 reads/sample for a given Blastocystis subtype.

Supplementary Table S5: Average prevalence and relative abundance of the different fungal species by sample type (.csv file)

Supplementary Table S6: Average prevalence and relative abundance of the different fungal species in the feces by country of origin (.csv file).

Supplementary Table S7: Average prevalence and relative abundance of the different 18S species by sample type (full dataset without mammalian blocking primer) (.csv file).

Supplementary Table S8: Average prevalence and relative abundance of the different 18S species in feces by country of origin (full dataset without mammalian blocking primer) (.csv file).

Supplementary Table S9: Average prevalence and relative abundance of the different 18S species by Sample Type (full dataset with mammalian blocking primer) (.csv file).

Supplementary Table S10: Average prevalence and relative abundance of the different 18S species in feces by country of origin (full dataset with mammalian blocking primer) (.csv file).

Supplementary Table S11: Metadata of the full ITS2 dataset (.csv file).

Supplementary Table S12: ASV table of the ITS2 dataset (.csv file).

Supplementary Table S13: Taxonomy table of the ITS2 dataset (.csv file).

Supplementary Table S14: Metadata of the full 18S dataset (.csv file).

Supplementary Table S15: ASV table of the 18S dataset without using a mammalian blocking primer (.csv file).

Supplementary Table S16: Taxonomy table of the 18S dataset (.csv file).

Supplementary Table S17: ASV table of the reduced 18S dataset using a mammalian blocking primer (.csv file).

Supplementary Table S18: Metadata of the reduced 18S dataset using a mammalian blocking primer (.csv file).

Supplementary Table S19: Comparison of microeukaryote-positive samples by microscopy and 18S rRNA sequencing

	With human blocker				No human blocker			
	**Madagascar (N=148)***		**Central African Republic (N=53)***		**Madagascar (N=261)***		**Central African Republic (N=173)***	
	Microscopy positive samples (%)	18S rRNA, >5 seq/sample (%)	Microscopy positive samples (%)	18S rRNA, >5 seq/sample (%)	Microscopy positive samples (%)	18S rRNA, >5 seq/sample	Microscopy positive samples (%)	18S rRNA, >5 seq/sample (%)
Ascaris spp.	75 (50.68%)	10 (6.76%)	0 (0%)	0 (0%)	137 (52.49%)	125 (47.89%)	1 (0.58%)	10 (5.78%)
Trichuris spp.	104 (70.27%)	26 (17.57%)	0 (0%)	0 (0%)	169 (64.75%)	102 (39.08%)	0 (0%)	0 (0%)
Enterobius spp.	4 (2.70%)	14 (9.46%)	0 (0%)	0 (0%)	7 (2.68%)	0 (0%)	0 (0%)	0 (0%)
Giardia spp.	34 (22.97%)	20 (13.51%)	9 (16.98%)	4 (7.55%)	52 (19.92%)	16 (6.13%)	39 (22.54%)	10 (5.78%)
Entamoeba spp.	35 (23.65%)	81 (54.73%)	11 (20.75%)	29 (54.72%)	49 (18.77%)	117 (44.83%)	39 (22.54%)	55 (31.79%)
Chilomastix spp.	1 (0.68%)	0 (0%)	2 (1.06%)	0 (0%)	2 (0.77%)	0 (0%)	7 (4.05%)	0 (0%)

*Samples with parasitological analysis using microscopy as well as 18S rRNA data
